# Evolutionary Tinkering with Conserved Components of a Transcriptional Regulatory Network

**DOI:** 10.1371/journal.pbio.1000329

**Published:** 2010-03-09

**Authors:** Hugo Lavoie, Hervé Hogues, Jaideep Mallick, Adnane Sellam, André Nantel, Malcolm Whiteway

**Affiliations:** 1 Biotechnology Research Institute, National Research Council, Montreal, Quebec, Canada; 2 Department of Biology, McGill University, Montreal, Quebec, Canada; 3 Department of Anatomy and Cell Biology, McGill University, Montreal, Quebec, Canada; Trinity College Dublin, Ireland

## Abstract

Gene expression variation between species is a major contributor to phenotypic diversity, yet the underlying flexibility of transcriptional regulatory networks remains largely unexplored. Transcription of the ribosomal regulon is a critical task for all cells; in *S. cerevisiae* the transcription factors Rap1, Fhl1, Ifh1, and Hmo1 form a multi-subunit complex that controls ribosomal gene expression, while in *C. albicans* this regulation is under the control of Tbf1 and Cbf1. Here, we analyzed, using full-genome transcription factor mapping, the roles, in both *S. cerevisiae* and *C. albicans*, of each orthologous component of this complete set of regulators. We observe dramatic changes in the binding profiles of the generalist regulators Cbf1, Hmo1, Rap1, and Tbf1, while the Fhl1-Ifh1 dimer is the only component involved in ribosomal regulation in both fungi: it activates ribosomal protein genes and rDNA expression in a Tbf1-dependent manner in *C. albicans* and a Rap1-dependent manner in *S. cerevisiae*. We show that the transcriptional regulatory network governing the ribosomal expression program of two related yeast species has been massively reshaped in *cis* and *trans*. Changes occurred in transcription factor wiring with cellular functions, movements in transcription factor hierarchies, DNA-binding specificity, and regulatory complexes assembly to promote global changes in the architecture of the fungal transcriptional regulatory network.

## Abstract 2

A surprising level of evolutionary plasticity is revealed by analysis of differences between related yeasts in the mechanisms regulating the essential cellular process of ribosomal gene expression.

## Author Summary

Conserved metabolic machineries direct energy production and investment in most life forms. However, variation in the transcriptional regulation of the genes that encode this machinery has been observed and shown to contribute to phenotypic differences between species. Here, we show that the regulatory circuits governing the expression of central metabolic components (in this case the ribosomes) in different yeast species have an unexpected level of evolutionary plasticity. Most transcription factors involved in the regulation of expression of ribosomal genes have in fact been reused in new ways during the evolutionary time separating *S. cerevisiae* and *C. albicans* to generate global changes in transcriptional network structures and new ribosomal regulatory complexes.

## Introduction

A conserved metabolic machinery forms the common basis of all cells; however, variation in the regulation of the genes that encode this machinery produces fundamental phenotypic differences between species. Recently, several groups have linked phenotypic traits to changes in the expression of conserved gene in diverse metazoans like Darwin finches, sticklebacks, and flies [1]–[6]. At the transcriptional level, this differential gene expression can be obtained by varying the structure of cellular transcriptional regulatory networks (TRNs), and many types of modifications can drive changes in gene regulation. For example, the set of target genes of a transcription factor (TF) can evolve by *cis*-regulatory sequence changes [7]–[19], as the appearance or disappearance of TF-binding motifs in genes or groups of genes allows their addition or removal from regulatory circuits. Changing the chromatin status of a gene by varying its nucleosome occupancy, its gene neighborhood, or its chromosome position can have impacts on its expression level [20],[21]. As well, *trans*-acting factors and their interacting partners can be modified by the recruitment of chromatin modifying enzymes or by changes in properties such as their DNA-binding specificity, modular structure, *trans*-activating potential, or combinatorial/cooperative binding characteristics [22]–[25]. Furthermore, the regulation of a TF can be changed through it being connected to new regulatory circuits, and this would affect the expression of its targets [19]. Recently, several studies have highlighted gene expression differences between species [26]–[28], but the flexibility of the regulatory network that drives these transcriptional changes still needs to be studied.

Ribosomal proteins (RPs) and rRNAs are among the most conserved components of the cell, and the transcriptional regulation required to produce their stoichiometric and condition-dependent expression is a central cellular process. In *S. cerevisiae*, co-ordinate expression of RP subunit genes is brought about by a protein complex made of the essential factors Rap1, Hmo1, Fhl1, and Ifh1. Rap1 and Hmo1 recruit the nutrient-dependent Fhl1-Ifh1 complex exclusively to RP genes [29]–[37], although Rap1 separately also occupies telomeres, the mating type locus, and glycolytic gene promoters [38],[39]. The binding of Rap1, Fhl1, and Hmo1 is not modulated by stress or nutrient levels, but under conditions of rapid proliferation, Fhl1 recruits Ifh1 through a heterotypic interaction between their respective FHA and FHB domains. This recruitment activates RP gene transcription to maximal levels but is perturbed by stress, or by inhibition of TOR or PKA signaling pathways, resulting in Ifh1 being released from RP promoters and replaced by another FHB-containing protein, the Crf1 co-repressor [31]. Therefore, in *S. cerevisiae*, the regulation of RP genes depends on intricate interactions among four regulatory proteins, specific DNA elements, and signaling pathways.

Previous studies have proposed that RP regulation has a high level of flexibility during evolution [7],[10],[15],[22]. This is supported by our recent observation that the essential *C. albicans* TF Tbf1 (assisted by Cbf1 at some loci) is the key DNA-binding regulator of RP genes and the rDNA locus in most fungal species [40]. Therefore, a Tbf1-DNA interface prevails at RP genes and the rDNA locus of *C. albicans*, while Rap1 governs the transcription of RP genes in *S. cerevisiae*. But the means by which the *C. albicans* Tbf1-dominated regulatory network performs the task of connecting ribosomal transcription with cellular signaling and the fate of the other *S. cerevisiae* regulators remains unknown. Here, we have used chromatin immunoprecipitation followed by microarray analysis (ChIP-CHIP) with full-genome coverage to show that conserved orthologous TFs can be profoundly repositioned within the regulatory network during evolution. Specifically, their regulons, their connections with cellular functions, their hierarchical position within the regulatory network, their DNA-binding specificity, and their assembly into higher order complexes are shaped during evolution.

## Results

### Regulons of Conserved TFs

We assessed the sequence conservation of all known RP regulators from several species in the *S. cerevisiae* and *C. albicans* phylogenic branches and found that Cbf1, Hmo1, Rap1, Ifh1, Fhl1, and Tbf1 have a readily assignable ortholog in both groups and at least one region in their protein sequence is highly conserved (Figure S1 and Text S1). However, a Crf1 ortholog could not be identified in the *C. albicans* clade, consistent with the recent appearance of this RP co-repressor in the fungal lineage and its strain-specific role in the budding yeast [35],[41]. The switch between an Ifh1-activated to a Crf1-repressed state is therefore unlikely to occur in *C. albicans*.

**Figure 1:**
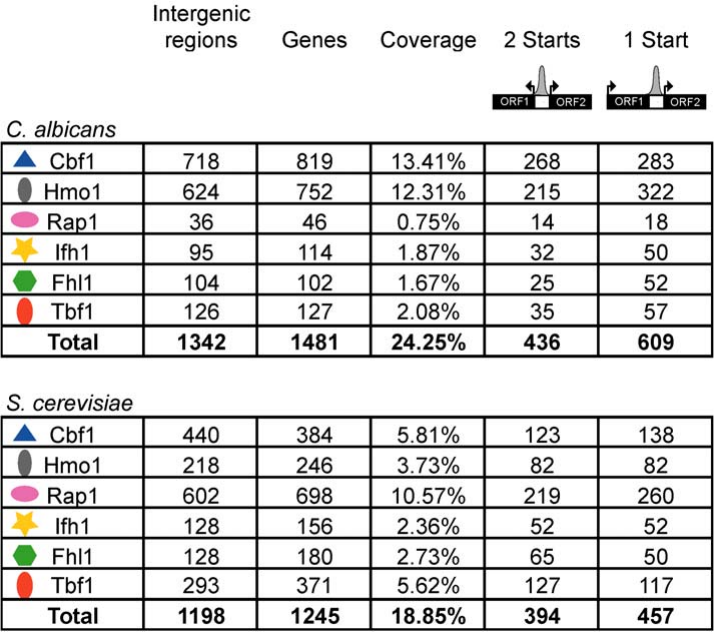
Summary of the transcription factor maps established in *C. albicans* and *S. cerevisiae*.

**Figure 2:**
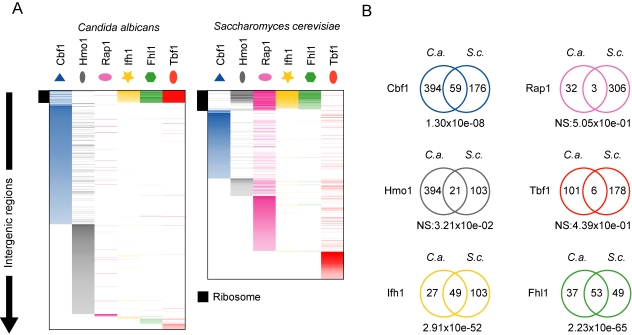
Evolution of the genome coverage of transcription factors involved in the RP transcriptional regulatory network. (A) Visual display of TF binding sites in the genomes of *S. cerevisiae* and *C. albicans*. Color saturation follows the log_2_ fold enrichment values in ChIP-chip experiments. Intergenic regions were first sorted by function (ribosome, sulfur starvation/amino acid biosynthesis, and glycolysis) and then by fold change for each regulatory protein. (B) Overlaps between the sets of targets of orthologous TFs of *C. albicans* and *S. cerevisiae*. The *p* values of each overlap were calculated using a hypergeometric distribution and is shown beneath each Venn diagram. NS stands for non-significant overlap.

We set out to determine the binding locations of tagged Cbf1, Hmo1, Rap1, Tbf1, Fhl1, and Ifh1 by ChIP-CHIP in haploid *S. cerevisiae* and diploid *C. albicans* strains (Table S1) with full-genome tiling arrays (20 and 17 probes/kb, respectively), and selected targets were validated by ChIP-qPCR (Datasets S1 and S2 and Figure S2). Although other regulators might or will be added to the list of RP TFs (like Sfp1 and the RGE; [15],[42]–[44]), the six factors studied here constitute the core of the characterized RP-specific regulatory network based on gene essentiality and *cis*-motif enrichment [10],[40]. Significant changes have occurred to the regulons bound by these TFs under rich growth conditions. First, their coverage (percent of the genome bound) has dramatically changed between species (Figures 1 and 2A) in a manner that is robust to the threshold used in the analysis of ChIP-CHIP data (Figure S3). The largest variations are the 10-fold reduced coverage of Rap1 in *C. albicans* and the 4-fold and 2.5-fold reduced coverage of Hmo1 and Cbf1 in *S. cerevisiae* (Figures 1 and 2A). As well, Fhl1, Ifh1, and Tbf1 in *S. cerevisiae* have roughly twice the number of target genes compared to their *C. albicans* orthologs (Figure 1). Second, the nature of the regulons changed: except for Cbf1, Ifh1, and Fhl1, which have maintained a significant proportion of their targets, Hmo1, Rap1, and Tbf1 have no significant overlap between the two species (Figures 2B and S3). Although binding of a TF in an intergenic region does not automatically have consequences on the regulation of the downstream ORF, all of the following analysis was conducted with the assumption that protein binding is at least a likely regulatory interaction and that statistical enrichment of TF targets within gene ontology (GO) categories is a good clue of a regulator's role within the cellular transcriptional network.

### Changes in Functional Connectivity of TFs

**Figure 3:**
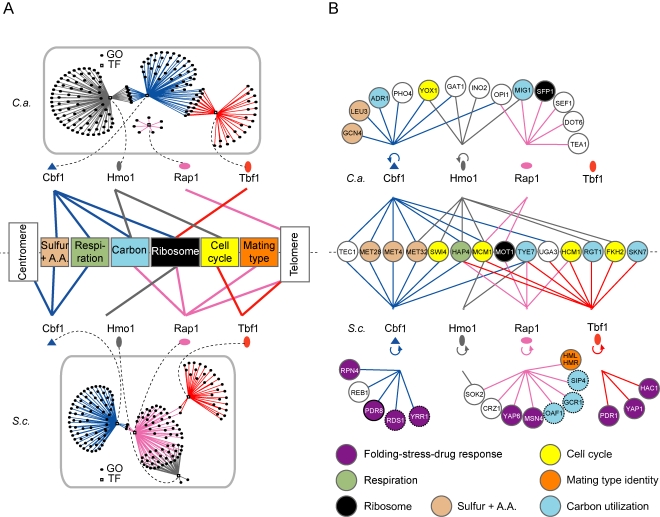
Functional involvement of generalist TFs of the RP transcriptional regulatory network has drastically changed between *S. cerevisiae* and *C. albicans*. (A) GO categories significantly enriched (*p*<1×10^−02^) in the target gene sets of each pleiotropic TF were displayed in a graph representing TF-GO interactions. A simplified representation of the regulatory interactions with major functional categories and chromosomal structures is presented between each species TF-GO interaction network. (B) Evolution of TF hierarchies between *S. cerevisiae* and *C. albicans*. A map of all significant regulatory relationships between each pleiotropic TF and the TFs present within each species cellular network was drawn. A dashed circle surrounds transcription factors uniquely found in *S. cerevisiae*.

Considering the high level of variation in the degree and distribution of transcriptional coverage of TFs, we tested if these changes impact on their connectivity with cellular functions. The targets of Cbf1, Hmo1, Rap1, Ifh1, Fhl1, and Tbf1 were systematically queried for their overlap with all GO categories. Two categories of TFs arise from this analysis: the generalist (Cbf1, Hmo1, Rap1, and Tbf1) and the specialist (Ifh1 and Fhl1) factors. Generalist TFs have connections with multiple functional categories in at least one of the two species, while the specialists are highly targeted to RP gene regulation in both *S. cerevisiae* and *C. albicans*. We will first focus on generalist factors and then describe how the interactions of specialists have been rearranged within the RP regulatory complex. To visually assess the shifts in functional connectivity, each GO category with a *p* value of enrichment smaller than 1×10^−02^ was considered connected to the generalist TF of interest and was displayed as a node in a TF-cellular function interaction network (Figures 3A and S4).

Cbf1 has a conserved role in the regulation of sulfur starvation and sulfur amino acid biosynthesis [40], and in both species it is bound upstream of one quarter of the respiratory-chain-coding genes (Figure 3A). In addition to these conserved functions, *C. albicans* Cbf1 specifically binds upstream of RP genes, at the rDNA locus, and upstream of glycolytic genes (*p* = 2.34×10^−05^; 46%; Figure 3A) [40]. As well, *S. cerevisiae* Cbf1 binds, as expected, to all centromeric regions (Figure S5), while its *C. albicans* homolog is totally absent from centromeres, consistent with the recent discovery of regional rather than “point” centromeres in *C. albicans* [7],[45]–[47].

Our *S. cerevisiae* Hmo1 ChIP-CHIP recapitulated the previously observed connection to the RP regulon, as well as some unrelated genes and its own promoter [34]. In contrast, *C. albicans* Hmo1 is essentially absent from the RP regulon (3 RP genes/752 target genes) but moderately recruited to genes involved in functions such as mono- and polysaccharide metabolism (glucose, fructose, glucan, and glycogen) (*p* = 2.98×10^−08^; GO:0019318), ergosterol metabolism (*p* = 7.00×10^−03^; GO:0006696), and cell cycle regulation (*p* = 4.13×10^−03^; GO:0051726) (Figure 3A).

*C. albicans* Tbf1 is exclusively located at RP gene loci as previously described, with all Tbf1 peaks with log_2_ enrichment ratios above 5-fold located in RP intergenic regions (72/72) [40]. In contrast, Tbf1 binding data in *S. cerevisiae* reveal that while it binds at only a few RP intergenic regions (5 RP genes/371 target genes), it is concentrated at sub-telomeric and telomeric regions (Figures 3A and S2) [48] as well as at 300 protein coding loci with moderate enrichments in GO categories related to cell cycle progression (*p* = 7.85×10^−03^; GO:0051726) and RNA polII TF activity (*p* = 2.25×10^−03^; GO:0003704) (Figure 3A), together with a subset of genes involved in rRNA processing (Sellam et al., manuscript in preparation). This suggests that *S. cerevisiae* Tbf1 is a generalist transcriptional regulator that transited from the specialist state following its replacement by Rap1 in the RP regulon.

*S. cerevisiae* Rap1 binds RP gene promoters (GO:0022626; *p* = 3.03×10^−80^), glycolytic enzyme promoters (GO:0006096; *p* = 8.98×10^−06^), the silent mating type locus, and the telomeres as reported (Figure 3A) [49],[50], while in *C. albicans* it binds none of the glycolytic genes and a single (*RPS5*) RP gene. Instead, *C. albicans* Rap1 binds to telomeres (Figure S2) and to a few (36) intra-chromosomal locations enriched upstream RNA polII transcriptional regulators (GO:0006357; *p* = 1.34×10^−03^). Altogether, apart from the connections of Cbf1 with sulfur starvation and respiration and of Rap1 with telomeric repeats, all edges in the functional network of these generalist TFs appear to have been reorganized between *S. cerevisiae* and *C. albicans*.

### Changes in Hierarchical Layers of the TRN

In addition to regulating coherent groups of functionally related structural genes, TFs can also act in hierarchical layers by controlling the expression of other TFs as well as key regulatory proteins like kinases or kinase regulators. Changing these hierarchies can have important functional consequences on cellular regulation, and therefore we examined the changes in generalist TF regulatory relationships within the networks obtained from our data. First, we found that TF auto-regulation (by feedback or feed-forward), commonly observed in regulatory network motifs [51]–[53] and detected here by the binding of a TF to its own promoter, could be gained or lost between species. While Cbf1 and Hmo1 bind their promoter region in both species, the probable auto-regulation of Rap1 and Tbf1 defined by protein binding is seen only in *S. cerevisiae* (Figure 3B). Second, the hierarchical layers of the TRN have been reorganized between species. The regulatory relationships between TFs appear to be plastic and the hierarchical status of TFs can change dramatically: for example, *S. cerevisiae* Tbf1 binds 11 TFs (*p* = 2.25×10^−03^) while it binds none in *C. albicans* (Figure 3B). Similarly, *C. albicans* Rap1 seems to have moved in the regulatory network hierarchy; six TFs rank in the 10 most Rap1-enriched intergenic regions in *C. albicans* while its *S. cerevisiae* homolog binds only 13 TFs amongst its 595 target genes (Figure 3B). Most interestingly, two of *C. albicans* Rap1-regulated TFs are Sfp1 and Dot6 (Figure S6) [42],[43],[54],[55], two master regulators of ribosomal biogenesis, as well as Mig1, a well-characterized glucose-responsive transcriptional repressor (Figure 3, S2, and S6) [56]. This suggests that the hierarchical status of Rap1 within the TRN has drastically changed in the yeast phylogeny. Altogether, only seven out of 68 connections in the TF regulatory network had been maintained between species.

Another well-studied example of transcriptional control loops is the temporal regulation of the cell cycle machinery [57]. We noted above that *C. albicans* Hmo1 and *S. cerevisiae* Tbf1 share a functional connection with the regulation of cell cycle progression. More precisely, *S. cerevisiae* Tbf1 binds the intergenic regions of the cyclins Cln1, Cln3, and Pcl2 and the cell cycle TFs Hcm1, Fkh1, and Mcm1, while *C. albicans* Hmo1 binds the kinase Swe1, the cyclin-dependent kinase Cdc28, the cyclins Cln3, Pcl5, and Pcl2, as well as the TFs Yox1, Mcm1, Swi4, and Fkh1 (Figures 3B and S6), but none of these regulatory interactions are observed for their respective orthologs. Signaling networks often impinge and rely on transcriptional regulators to promote a cellular response. When a systematic survey of the TF-kinase network is conducted, only two of the 52 total connections are conserved between *S. cerevisiae* and *C. albicans* (Figure S7). Thus, in addition to the rewiring of structural metabolic gene circuits, major modifications in hierarchical regulatory relationships can be observed within the transcriptional network.

### Changes in TF DNA-Binding Specificities

**Figure 4:**
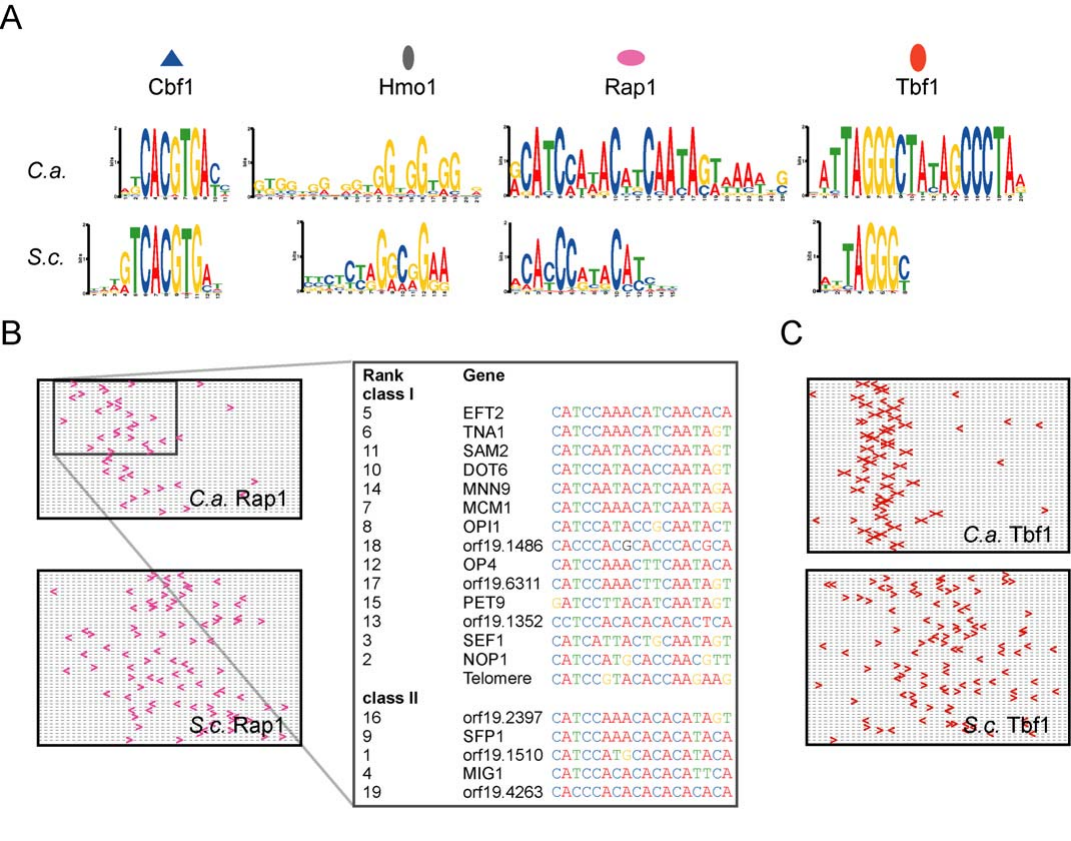
Evolution of the DNA-binding specificities of generalist TFs. (A) De novo prediction of DNA elements bound by each pleiotropic TF in each species with MEME [122]. (B) Representation of the positions and orientations of Rap1-bound elements in target promoters. Loci bound by *C. albicans* Rap1 yield a 16 bp consensus sequence. (C) Tbf1 has a strict requirement for a TTAGGGN6CCCTAA palindrome in *C. albicans* while TTAGGG motifs with random spacing and orientation are required for the binding of *S. cerevisiae* Tbf1.

We next examined the DNA-binding specificities of the rewired generalist TFs. Apart from Cbf1 that has maintained its DNA-binding specificity (tCACGTGa), the consensus sequence bound by Hmo1, Rap1, and Tbf1 varies between species. Our analysis of *S. cerevisiae* Hmo1 yielded the previously described IFHL motif with a strong CTAGGCGG consensus (*E*-value = 5.9×10^−14^) (Figure 4A) [34]. Interestingly, *C. albicans* Hmo1 is strongly associated with a GGT repeat motif forming the GGTGGTGG consensus (*E*-value = 6.7×10^−172^), and thus the two orthologous TFs share a GGYGG consensus sequence, suggesting that the portion contacted by Hmo1 in both species is made of repeats of GGY_(n)_. The TF Rap1 has a well-defined specificity for the CACCCNNACA motif in *S. cerevisiae* that we retrieved from our full-genome binding data (Figure 4A) [58],[59]. On the other hand, *C. albicans* Rap1 seems to have a more specific interaction with DNA at the CATCCANACANCAATAG motif in a threshold robust manner (*E*-value = 1.8×10^−32^)(Figures 4A, 4B, and S9) consistent with a recent analysis of *C. albicans* Rap1 specificity [60]. Interestingly, the telomeric DNA of *C. albicans* consists of repeats of the 23 bp telomeric RNA sequence (encoded on chromosome R), and the junction of two of these repeats (CATCCGTACACCAAGAA) matches 11 of the 15 bp of this consensus (Figure 4B) [61]. Therefore, many changes in the telomeric RNA-coding gene, in the intergenic region of Rap1 target genes, and in the protein sequence of the Rap1 Myb DNA-binding domain have co-evolved [60],[62]. Finally, the DNA motif bound by Tbf1 in *S. cerevisiae* is limited to several clustered occurrences of the TTAGGG motif (*E*-value = 1.8×10^−35^) with no requirements of orientation or spacing (Figure 4A and 4B), and therefore it does not have the tight association with an 18 bp palindrome as seen in *C. albicans* (Figure 4A and 4B).

### Evolution of a TF Assembly Connected to Cellular Signaling

**Figure 5:**
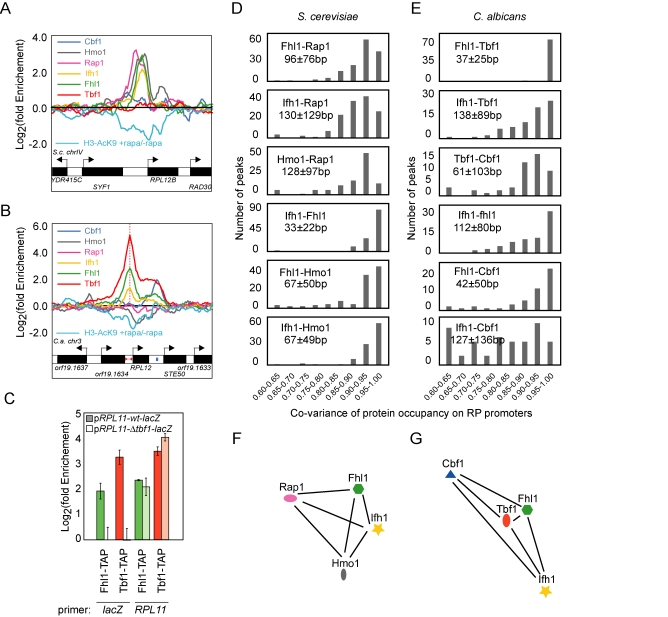
The RP regulatory complex of *C. albicans* is assembled differently than in *S. cerevisiae*. Assembly of the RP-specific TFs at *S. cerevisiae* (A) and *C. albicans* (B) *RPL12* intergenic regions as determined by ChIP-CHIP on full-genome tiling arrays. The rapamycin-dependent acetylation of Histone H3 Lysine 9 is also displayed (cyan profile). (C) Tbf1-dependent binding of Fhl1 at the RP chimeric construct *pRPL11*::*lacZ*. Pairwise analysis of the co-variance of ribosomal transcription factors binding profiles on RP promoters in *S. cerevisiae* (D) and *C. albicans* (E). Average distances (in bp) between *S. cerevisiae* Rap1 maximum peaks of enrichment and the Hmo1, Fhl1, and Ifh1 peaks and between *C. albicans* Tbf1 peaks and the Cbf1, Fhl1, and Ifh1 peaks at RP promoters are displayed. Graph representing the average distance between ribosomal transcription factors weighted on their co-variance at RP promoters in *S. cerevisiae* (F) and *C. albicans* (G).

Fhl1 and Ifh1 are the only TFs that conserved their function in ribosomal regulation between *C. albicans* and *S. cerevisiae*; functional analysis of their genes with tetracycline repressible alleles and of their gene products by yeast-two-hybrid and co-immunoprecipitation supports that they are essential RP regulators forming a conditional FHA-FHB heterodimer (Figures S10 and S13 and Text S1), and in *C. albicans*, all peaks common to Tbf1, Fhl1, and Ifh1 occur upstream of RP genes and the rDNA locus (Figures 2A and S11). In *S. cerevisiae*, Rap1, Hmo1, Fhl1, and Ifh1 binding also co-occur on RP intergenic regions, and it is well established that Fhl1 and Ifh1 recruitment relies on neighboring Rap1 and Hmo1 binding (Figure 5A) [30],[34],[36]. Since Rap1 and Hmo1 are absent from *C. albicans* RP promoters, we wondered whether Tbf1 is required for tethering Fhl1 and Ifh1 to RP promoters. For this, we analyzed Tbf1 and Fhl1 binding to a *RPL11* promoter (p*RPL11*) containing (p*RPL11-wt-lacZ*) or devoid (p*RPL11-Δtbf1-lacZ*) of the *tbf1* element [40],[63]. Deletion of the *tbf1* palindrome caused dissociation of both Tbf1 and Fhl1 from the *lacZ* chimera (primer: *lacZ*), while binding was normal on the remaining wild-type *RPL11* locus (primer: *RPL11*) (Figure 5C). This confirms that *C. albicans* Tbf1 is required for Fhl1-Ifh1 recruitment.

The fact that the binding of Fhl1 is Rap1 and Hmo1-dependent in *S. cerevisiae* and Tbf1-dependent in *C. albicans* raises the question of the evolution of the ribosomal regulatory complex. To better characterize the properties of these distinct TF assemblies, we interpolated the signal ratios of our tiling array data to each individual base pair and evaluated the co-variance of TF occupancies by calculating the pairwise scalar product of signal intensities along RP promoter regions for each pair of TFs. This detailed, multivariate analysis shows that not only the players within the RP regulatory complex have changed but that their positioning with respect to each other has also evolved. The most striking difference is the strict co-variance of Fhl1 and Tbf1 occupancies in *C. albicans* compared with the more loose association of Fhl1 and Rap1 signals in *S. cerevisiae* (Figure 5D and 5E). This high-resolution numerical analysis of TF co-occupancies is corroborated by comparing the position of occupancy peaks between TFs (Figure S12). The peak coordinates of *C. albicans* Fhl1 occur at 37±25 bp from the Tbf1 peaks (Figure S12), while consistent with previous studies [30],[34],[35], Fhl1 and Rap1 are located 96±76 bp apart in *S. cerevisiae* (Figure S12). When considered from the perspective of the main DNA-binding regulators Tbf1 and Rap1, *C. albicans* Fhl1 and Ifh1 therefore display different binding patterns compared to their *S. cerevisiae* counterparts. This suggests that Tbf1, Fhl1, Ifh1, and Cbf1 form a complex through distinct interactions and in a distinct cooperative mode in *C. albicans* compared to the Rap1-Hmo1-Fhl1-Ifh1 complex of *S. cerevisiae*.

Finally, we wanted to establish if this remodeled RP regulatory complex had conserved its connections with cellular signaling and chromatin modifications. For this, we first confirmed that the expression of ribosome subunits is TOR and PKA sensitive in *C. albicans* and, therefore, it is connected to signal transduction pathways known to affect *S. cerevisiae* RP gene transcription (Figure S10 and Text S1) [64]. Accordingly, the formation of the Fhl1-Ifh1 heterodimer is both signal- and stress-dependent in *C. albicans*, as was observed in *S. cerevisiae* (Figure S13). We then asked whether similar histone modifications act downstream of the ribosomal TFs in *S. cerevisiae* and *C. albicans*. For this, we carried out genome-wide analysis of acetylated histone H3 lysine 9 (H3-AcK9) before and after treatment with rapamycin (Figure 5; cyan-colored line labeled as H3-AcK9 +rapa/−rapa). We detected significant decreases in the acetylation profile of 143 *S. cerevisiae* ORFs and 74 *C. albicans* ORFs, of which 73% and 60%, respectively, are RP genes (Figures 5A–B and S11). Clusters of rapamycin-sensitive H3-AcK9 modifications near the RP genes start sites are thus conserved in *C. albicans* and *S. cerevisiae* (Figures 5A and 5B and S11) [65]. This demonstrates that the signaling-dependent Ifh1 association, histone modifications, and probably the recruitment and dissociation of the histone acetylation/deacetylation machinery at RP promoters occur in both species despite the remodeling of the ribosomal TF complex [66]–[69].

## Discussion

Flexibility of TRNs is essential to promote new adaptations and conditional utilization of the conserved metabolic machinery. The rewired ribosomal regulon of *S. cerevisiae* and *C. albicans* constitutes an ideal model to assess how gene regulatory circuits evolve to generate new network structures. Here, we have shown that conserved components of the essential RP TRN were reused for different purposes in two related fungi.

### Connection and Disconnection of Cellular Functions by *Cis*-Regulatory Turnover

While the three-dimensional structure of TFs is relatively constrained, intergenic DNA is intrinsically plastic. The addition or removal of DNA sequences by point mutations has little impact on the overall structure of the DNA molecule, whereas protein structures are less tolerant to non-synonymous changes. In addition, promoter modifications can provoke changes in gene expression without the pleiotropic effects caused by modifications to *trans*-acting factors [70]. The addition of target genes to a regulon by *cis*-regulatory motif turnover is thus intuitively the simplest change that can occur within a regulatory network and this allows the exploration of countless regulatory interactions with minor fitness cost. In the minimal regulatory network studied here, we observed massive *cis*-regulatory changes, the most prominent involving Cbf1, Hmo1, Rap1, and Tbf1.

One of the consequences of this *cis*-regulatory lability is that, at the functional level, DNA-binding TFs can shuttle between general and highly specialized regulatory functions by *cis*-regulatory motif turnover. We speculate that a repertoire of generalist TFs like Cbf1, Hmo1, Rap1, and Tbf1 is maintained within cells and might normally serve a yet undefined role. Otherwise, these readily available DNA-binding cassettes can be recruited to new cellular functions and regulons by the appearance of *cis*-regulatory motifs in promoter regions without dramatic detrimental effects and without the need for complex structural changes in their DNA-binding specificity.

**Figure 6:**
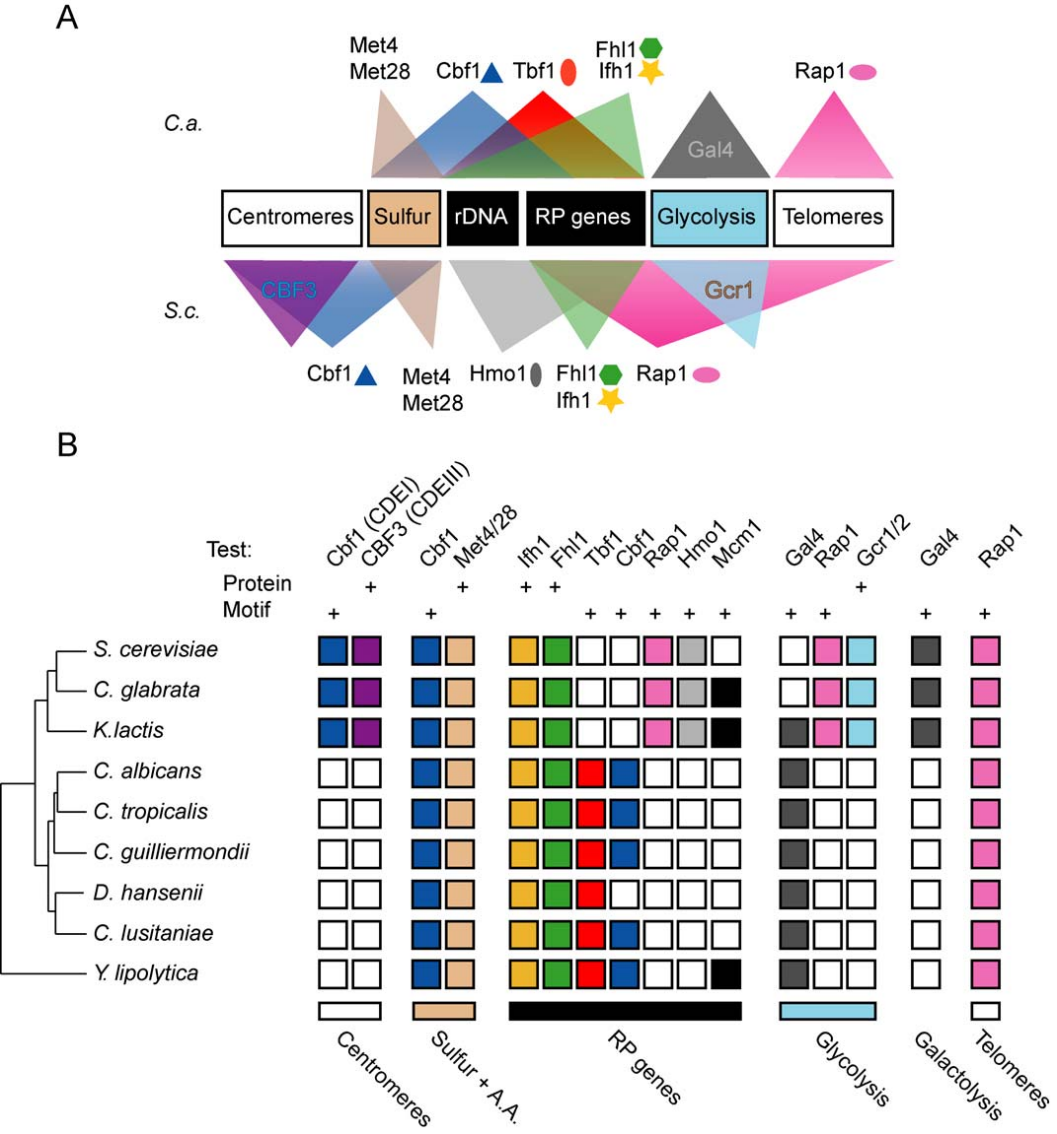
Evolution of the ribosomal transcriptional regulators of fungi. (A) Regulons and chromosomal structural elements are bound by combinations of transcription factors and co-factors that evolved between *C. albicans* and *S. cerevisiae*. (B) Simplified representation of motif enrichment and protein complex conservation at the different loci and biological functions bound by RP regulators in nine hemiascomycetes species. Motif enrichments were previously described [40]. Phylogenetic profile of protein conservation was derived from a recent analysis of gene conservation in the ascomycetes lineage [41].

Another consequence of these *cis*-regulatory changes is the direct coupling of regulons through the binding of a single TF. For example, the recruitment of Rap1 at the RP, glycolytic, and telomeric regulatory complexes through numerous *cis*-regulatory changes in *S. cerevisiae* associated these three regulons and most likely promoted their co-regulation (Figure 6A). By contrast, in *C. albicans* these regulons appear more insulated, with glycolysis being regulated by the TF Tye7 assisted by Gal4 [71]–[73]. A similar assumption can be made for the connection of the RP, electron transport chain, and sulfur starvation regulons mediated by *C. albicans* Cbf1 and for the relationship between cytosolic and mitochondrial RP genes in *C. albicans* [15]. A recent meta-analysis of gene expression profiles showed that indeed the coupling of RP genes with various regulons, including energy derivation pathway genes, is different in *S. cerevisiae* versus *C. albicans* [20]. The species-specific connections of regulons by *cis*-regulatory motif turnover observed here most likely accounts for this evolvable co-regulation of RP genes with other coherent gene sets. These TF-mediated links between cellular functions very likely specify distinct physiological responses between species.

At a level above the simple control of metabolic regulons and circuits of structural genes, authors have suggested the existence of kernels and hierarchies regulating complex temporal and spatial decisions during growth and development [74]–[77]. Here, we show that TF hierarchies can drastically evolve; structural gene regulators like *C. albicans* Tbf1 and *S. cerevisiae* Rap1 can move up and down the hierarchies of the transcriptional network and become regulators of other transcriptional switches. For example, Rap1, instead of directly binding the structural genes of the ribosome and glycolysis as seen in *S. cerevisiae*, regulates their regulators Sfp1, Dot6, and Mig1 in *C. albicans*. It has been speculated that, in metazoans, such changes are precluded by the complexity of highly interconnected circuits [75] even though examples of hierarchical flexibility like the addition of fog-2 in the sex-determination pathway of worms and the insertion of bicoid in the anteroposterior patterning pathway of flies argue against this rigidity of developmental pathways [78]–[82]. Nevertheless, it appears that TFs within ascomycete regulatory networks can move vertically in the hierarchies of the TF-TF network [19]. This also suggests that the hierarchical organization of the regulatory network is often exploited to generate gene expression diversity in unicellular organisms. All of the above observations support the idea that intergenic sequences explore various possible regulatory relationships permitting drifts (Cbf1) or complete changes (Hmo1, Rap1, and Tbf1) in the regulons controlled by conserved generalist TFs.

### *Trans*-Regulatory Changes and Differential TF Assemblies

In addition to changes in *cis*-regulatory sequences, several *trans*-acting factor modifications were required to generate a new transcriptional network structure. These are suggested by the changes in the primary sequence of orthologous regulatory proteins (Figure S1), by the distinct DNA specificities of orthologous *trans*-acting factors (Figure 4), and by the creation of new TF assemblies between the two species under study (Figures 5 and 6). Our findings thus support recent observations that *cis*- and *trans*-acting mutations must co-occur in the evolution of gene expression differences [24],[26],[70],[83],[84].

Cooperative and combinatorial TF assemblies are essential to maximize the use and focus the action of general transcriptional regulators by insulating or compartmentalizing their different functions [22],[85]. And although the appearance of new TF assemblies involves complex structural changes, these occurred at many loci between *S. cerevisiae* and *C. albicans*. In fact, Cbf1, Hmo1, Rap1, and Tbf1 all have acquired or lost function-specific interactions with other TFs between *S. cerevisiae* and *C. albicans*: the interactions Cbf1-CBF3, Rap1-Hmo1-Fhl1-Ifh1, and Rap1-Gcr1/2 are exclusive to *S. cerevisiae*, while the Cbf1-Tbf1-Fhl1-Ifh1 association is uniquely seen in *C. albicans* [38],[49],[86]–[88]. Accordingly, the proteins contacting Cbf1 (CDEI-binding protein) within the centromeric CBF3 (CDEIII-binding) complex and the highly structured sequence of CDEI and CDEIII elements at the point centromeres are exclusively found in the *S. cerevisiae* lineage while other sequenced hemiascomycetes like *C. albicans*, *Y. lipolytica* and *D. hansenii* have regional centromeres with no sequence conservation and do not share orthologs of the CBF3 subunits (Figure 6B) [41],[47],[88]–[92]. Similarly, the glycolytic TFs Gcr1 and Gcr2 are only found in *S. cerevisiae* and close relatives (Figure 6B) [41]. The data presented here also support that the interfaces between orthologous proteins have been remodeled to form distinct ribosomal regulatory complexes composed of Rap1, Hmo1, Fhl1, and Ifh1 in *S. cerevisiae* and of Tbf1, Fhl1, and Ifh1 in *C. albicans*. This species-specific formation of complexes involved in gene regulation or chromosome maintenance is reminiscent of observations made on the pleiotropic regulator Mcm1, in particular its recruitment in close proximity with Rap1 consensus sites at RP gene promoters of *K. lactis* (Figure 6B) [22].

The structural basis of these combinatorial interactions on promoters remains unknown and the mechanism responsible for their appearance is unsolved, but a plausible scenario is that the law of mass action and the colocalization of proteins on the DNA and chromatin scaffolds favors TF-TF contacts, promoting the assembly of distinct regulatory complexes with different functional features between species [22],[93]. It is possible that higher order chromosomal or nuclear structures (like centromeres, telomeres, or chromosome territories) increase the effective concentration of transcriptional regulators and drive the association of target genes with DNA-binding proteins and the formation of new TF assemblies. This hypothesis is consistent with the convergent cooption of telomere-binding TFs in the regulation of RP genes [40], with the association of some TFs with specific chromosomal loci [21],[94]–[96], and with the fact that DNA-binding proteins involved in telomeric and centromeric maintenance appear highly evolvable in ascomycetes [47],[60],[62].

### A Selective Pressure from Inside the Regulon

In addition to ribosomal regulation, recent work has shown that the transcriptional control of the glycolytic regulon has also experienced major changes in the evolution of fungi [73],[97]. Since glycolysis and ribosome synthesis are both essential determinants of fitness, a requirement for metabolic coherence probably synchronizes the fixation of these dramatic *cis*- and *trans*-regulatory changes. Regulons are, by definition, groups of genes that need to be co-expressed in order to maintain the stoichiometry of protein complexes or the flux of metabolic pathways. The rate of fixation of regulatory changes is thus likely to correlate with the fitness consequence of altering the co-expression of a given regulon.

*In silico* simulations of phenotype accessibility from diverse genotypes have shown that adaptive progress coincides with discontinuous structure transformations [98]. In this system, sudden phenotypic changes in RNA secondary structure were preceded by extended periods of neutral genotypic drift, and the system was primed for adaptive changes by neutral mutations that allow these phenotypic transitions [98]. We think that such a combination of neutral and adaptive processes can be evoked to explain the massive regulatory network rewirings described here. In a first phase, the ribosomal TRN is under strong selection and could only drift by the accumulation of neutral mutations to produce a new genotypic *cis*-regulatory context for the fixation of a new network structure. In the second phase, changes in the transcriptional regulation of one or a few RP subunits by *cis*-regulatory variation would cause an imbalance in the stoichiometry of the ribosome and a rapid correction would necessarily follow, leading to a discontinuous change in the nature of the regulatory circuit underlying RP co-expression. It is possible that only configurations where a dominant *cis*-regulatory element ensures subunits co-expression, as observed for the *S cerevisiae* and *C. albicans* RP regulatory circuits, provide the required system stability and that intermediate more complex network organizations are unstable and transitory. Therefore, based on this hypothesis, once committed on a new regulatory trajectory, a group of genes for which co-regulation is mandatory would reconverge to a new stable regulatory program. Metaphorically, these spectacular bifurcation events can correspond to the similar behavior of simulated non-linear dynamic systems or to punctuated equilibria and could be characterized by the same directionality, irreversibility, and modularity [98]–[102]. These observations raise the question of whether the phenomena described here are specific to highly expressed and co-regulated genes or can be observed in systems not requiring a high degree of co-expression. Also, whether TF rewiring and substitutions are driven by ecological pressures and if initial *cis*- and *trans*-regulatory mutations accumulate under selective pressure or under neutrality remain open questions.

Finally, the connection of cellular signaling pathways (through the Fhl1-Ifh1 complex) with downstream regulatory events, such as histone modifications, is conserved between species despite the substitution of the main DNA-binding module of the RP regulatory complex. DNA-binding TFs and the regions they associate with might thus be the most interchangeable parts of a machine conveying external stimuli to the transcriptional apparatus. Tinkering with components of the transcriptional regulation of metabolic machines must therefore obey to both internal and external demands and is likely subjected to some selective pressure.

### Conclusion

Extensive as well as more limited transcriptional rewirings have been predicted for prokaryotic regulatory circuits [85],[103]–[107] and for ascomycetes transcriptional networks [7],[10],[15], but few comprehensive experimental validations are available for these systems [19],[22],[108],[109]. This investigation provides a full-genome comparative map under rich growth conditions of a central regulatory network that has experienced important changes. Overall, the machinery executing the ribosomal expression program of two related yeast species has been dramatically reshaped in *cis* and *trans*. The changes observed include transitions from the control of general to highly specialized regulons, rewiring to distinct cellular functions and to chromosomal structural elements (centromeres and telomeres), modifications of the hierarchical position of TFs within the regulatory network, modifications in TF DNA-binding specificities, and the remodeling of TF assemblies. This reutilization of conserved TFs at RP promoters was thus accompanied by large-scale changes in the architecture of the fungal TRN. This reorganization of the ribosomal gene expression circuitry thus constitutes a striking example of evolutionary tinkering.

## Materials and Methods

### Strains, Media, and Plasmids

Cell growth, transformation, and DNA preparation were carried out using standard procedures [110]. ChIP-CHIP experiments were conducted in the BWP17 strain background and the tetracycline titratable alleles of *CBF1*, *HMO1*, *IFH1*, *FHL1*, and *TBF1* were generated in the CAI4 background [111]. Cells were grown at 30°C. Synthetic dextrose was SD-Ura, SD-His-Arg, SD-Trp-Leu-Ade, -Trp-Leu-His, or -Trp-Leu (0.67% Yeast Nitrogen Base, 2% glucose, amino acids drop-out), and rich medium was YPD (1% Yeast Extract, 2% peptone, 2% dextrose). When stated, tetracycline was added to a concentration of 100 ug/ml for the indicated time [111].

The Tbf1-TAP and Cbf1-TAP constructs were previously reported [40]. Hmo1, Rap1, Fhl1, and Ifh1 were in vivo TAP-tagged with a TAP-*URA3* PCR product containing 100 bp homology up and downstream of each ORF and transformed in the *C. albicans* BWP17 background [63]. The doubly tagged Fhl1-HA/Ifh1-Myc strain was generated with a similar procedure with HA-*HIS1* and Myc-*ARG4* PCR products [63]. Correct integration of the tags was verified by PCR and sequencing. The *pRPL11-wt-lacZ* and *pRPL11-Δtbf1-lacZ* chimeras were reported elsewhere [40],[63]. C-terminal TAP-tag fusions of *S. cerevisiae* Cbf1, Tbf1, Ifh1, and Hmo1 were obtained from Open Biosystems [112], and the N-terminal TAP-Rap1, the Fhl1-HA, and Fhl1-HA/Ifh1-Myc tagged strains were kindly provided by Dr. Jonathan Warner [33]. The genotypes of all strains are listed in Table S1.

Protein interaction assays were done with fragments of Fhl1 and Ifh1 cloned by PCR in the Yeast-two hybrid plasmids pGADT7 and pGBKT7 between SfiI and XmaI (Clontech Laboratories Inc.). pGADT7 and pGBKT7 plasmids were then transformed in the haploid yeast strains pJ69-4a or pJ69-4α, respectively [113]. Diploids carrying both plasmids were created by mating on YPD followed by selection on SD-Trp-Leu, SD-Trp-Leu-His, or SD-Trp-Leu-Ade.

### Microarray techniques

ChIP experiments were performed as described previously with some modifications [114]. Briefly, cells were grown to an optical density at 600 nm of 0.6 in 50 ml of YPD. We followed the ChIP protocol available at http://www.ircm.qc.ca/microsites/francoisrobert/en/317.html with the following exceptions: chromatin was sonicated to an average 300 bp and 700 ul of whole-cell extract (WCE) were incubated with IgG-sepharose (GE Healthcare), anti-HA (12CA5), or anti-H3K9 antibody (Millipore, 06-942) adsorbed to protein G sepharose (GE Healthcare) [40],[63]. Immunoprecipitated DNA was used for either whole-genome location profiling or gene-specific real-time quantitative PCR analysis. For whole-genome location profiling, tagged ChIPs were labeled with Cy5 dye and untagged (mock) ChIPs were labeled with Cy3 dye. Probes were either hybridized to our *C. albicans* whole genome microarrays [63], custom *C. albicans* tiling arrays, or *S. cerevisiae* tiling arrays (Agilent Technologies). Microarray hybridization, washing, scanning, and normalization were performed as described [115]. Precise peak location and detection from normalized tiling array data was performed by (1) applying a Gaussian blur to log_2_ fold enrichment ratios, (2) interpolating oligonucleotide probe data to each base pair by a natural spline function, and (3) defining discrete peaks by sequentially extracting the most intense 1 bp peaks and masking a neighboring window of 600 bp (10 probes). Significant peaks were defined as having an enrichment value superior by at least two standard deviations (Z score = 2.0) to the mean of raw probe fold enrichment distributions defined individually for each experiment. The justification for this threshold can be found in Text S1 and is substantiated in Figures S3 and S4. Significantly bound regions for each factor and in each species are provided as supplementary data (Datasets S1 and S2). Gene expression profiling by microarray was performed as described previously [40].

### Co-Immunoprecipitations

*C. albicans* Fhl1-HA/Ifh1-Myc or *S. cerevisiae* DH36 [33] cells were grown to mid-log phase (an OD_600nm_ of 0.7–0.8) and exposed to various treatments: 1 ng/ml rapamycin for 30 min, heat shock at 46°C for 1 h, osmotic shock (OS) with 0.5 M of sodium chloride in YPD for 30 min, and hypoxia in oxygen-depleted YPD from 0.1 OD600nm to mid-log phase in sealed flasks. Cells at a final OD_600nm_ of 1.0–1.5 were harvested by centrifugation and lysed by bead beating in IP150 buffer (50 mM Tris-HCl [pH 7.4], 150 mM NaCl, 2 mM MgCl2, 0.1% NP40). The lysates were then cleared by centrifugation and protein concentration was estimated using the Bradford assay. One mg of total protein was added to 40–50 ul of monoclonal mouse anti-Myc (9E10) or anti-HA (12CA5) conjugated beads (Roche) and incubated at 4°C with end over end movement overnight. The next morning, beads were spun down at 4,000 rpm at 4°C, washed 3 times with IP150 buffer, boiled with SDS-PAGE loading buffer, and resolved on a 4%–20% gradient SDS-PAGE. Proteins were transferred onto a nitrocellulose membrane, blocked in 10% milk in PBST for 1 h at room temperature, and exposed to rabbit Anti-Myc (1∶1000) (Santa Cruz) or Anti-HA (1∶2500) (Roche) antibody overnight at 4°C. The membranes were finally hybridized with a goat-anti-rabbit HRP (1∶5000) (Thermo Scientific) and revealed with the Lumi-light Western Blotting Substrate (Roche).

### Quantitative PCR (qPCR)

Quantitative real-time PCR was performed using the Corbett Rotor-Gene RG-3000A (Corbett Research, Sydney, Australia) with SYBR Green fluorescence (Qiagen). Real-time PCR was performed using 1 ng of ChIPed DNA or total genomic DNA extracted from WCE. Cycling was for 15 min at 95°C, followed by 45 cycles of 95°C, 10 s, 56°C, 15 s, and 72°C, 15 s. All samples were tested in triplicate and means were used for further calculations. Fold enrichments of tested promoter sequences were estimated by using the coding sequence of the *C. albicans ACT1* ORF as a reference.

### RNA Species Distributions

Electropherograms of RNA species distributions were obtained by capillary electrophoresis with fluorescence detection on an Agilent Bioanalyzer 2100 (Agilent Technologies). Bioanalyzer RNA 6000 Nano Chips (Agilent Technologies) were loaded with 250 ng of total RNA before and after treatment with tetracycline by following the manufacturer's protocol.

### Informatics and Statistics Procedures

Multiple sequence alignments were performed with clustalX (http://www.embl.de/~chenna/clustal/darwin/) [116] and edited by using Seaview (http://pbil.univ-lyon1.fr/software/seaview.html) [117]. Phylogenetic trees derived from sequence alignments were produced with the PHYLIP package [118]. All hierarchical clustering and heatmap displays of the sequence alignments, ChIP-CHIP, or expression profiling data were done with the Cluster and Treeview programs (http://rana.lbl.gov/EisenSoftware.htm).

For motif detection, a DNA sequence corresponding to a 300 bp window centered on each tiling array peak was extracted. The sequences corresponding to the highest quarter of signal ratios for each TF were submitted to the MEME online server (http://meme.sdsc.edu/meme4/cgi-bin/meme.cgi) [40],[119]. The motifs uncovered by MEME were subsequently validated with randomized sets of target genes of equal size for each TF (Figure S9 and unpublished data). Our mini-motif detection algorithm was also applied to the same sets of 300 bp sequences on the full data set for each TF. Briefly, to uncover enriched *cis*-regulatory elements, every mini-motif composed of two nucleotide triplets separated by less than 16 bp (XXXn_(0–15)_XXX) was tested for its over-representation in peak regions compared to its occurrence in a randomized sequence space of equal size. Sequences possessing the mini-motif were defined as those with at least one instance of the motif or its reverse complement in their upstream region. The enrichments were calculated using a hypergeometric distribution.

Orthology tables relating *S. cerevisiae* and *C. albicans* genes were derived from the *Candida* genome database (CGD; http://www.candidagenome.org) and the *Saccharomyces* genome database (SGD; http://www.yeastgenome.org/) as well as http://www.broad.mit.edu/regev/orthogroups/. GO annotations of *C. albicans* and *S. cerevisiae* were obtained from CGD and SGD. Gene's GO annotations were systematically expanded to include the GO terms hierarchical relationships obtained from http://www.geneontology.org/. These annotations were used as queries for the sets of targets of each TF. GO enrichments in each set of TF-bound target genes were calculated with the hypergeometric distribution [120]. The significance threshold was set at *p*<10^–4^ with a randomized set of GO categories of equal size, and we used a conservative threshold of *p*<10^–2^ in the analysis of the TF-GO network.

The lists of TFs and kinases used to generate the TF-TF and TF-kinase networks presented in Figures 3 and S7 were obtained from the YEASTRACT database (http://www.yeastract.com/) [121] and kinase.com (http://kinase.com/scerevisiae/yeastkinase.htm), respectively. These lists were limited to the set of genes that share orthologs in *S. cerevisiae* and *C. albicans* and then intersected with lists of TF target genes from our ChIP-CHIP data using Microsoft Access.

The TF-GO, TF-TF, and TF-kinase networks represented in Figures 3 and S7 were produced using Pajek (http://pajek.imfm.si/doku.php) and the raw flatfiles were converted to Pajek format with Excel2Pajek (http://vlado.fmf.uni-lj.si/pub/networks/pajek/howto/excel2Pajek.htm).

## Supporting Information

Dataset S1 **Targets of** ***C. albicans*** **Cbf1, Hmo1, Rap1, Fhl1, Ifh1, and Tbf1 transcription factors determined by full-genome tiling arrays.**
(1.09 MB XLS)

Dataset S2 **Targets of** ***S. cerevisiae*** **Cbf1, Hmo1, Rap1, Fhl1, Ifh1, and Tbf1 transcription factors determined by full-genome tiling arrays.**
(1.04 MB XLS)

Figure S1 **Variation in the primary sequence and domain organization of orthologous TFs.** Alignment similarity maps of orthologs of the transcription factors (TFs) Cbf1, Hmo1, Rap1, Tbf1, Fhl1, and Ifh1 involved in the ribosomal protein (RP) transcriptional regulatory network of *S. cerevisiae* or *C. albicans*. *C.a.* and *S.c.* stand for *C. albicans* and *S. cerevisiae* and were used in all figures. Shading of the alignments reflects the percentage of conservation within the *C. albicans* (*C. albicans*, *Pichia stipitis*, *Debaryomyces hansenii*, and *C. guilliermondii*) or the *S. cerevisiae* (*S. cerevisiae*, *Ashbya gossipii*, and *Kluvyeromyces lactis*) branches or between the two branches. Histograms reflect the average phylogenetic distance derived from the PHYLIP distance matrix within (intra-*S.c.* and intra-*C.a.*) or between (*C.a.*-*S.c.*) branches. Distances showing a significant difference (*p*<0.01) compared to the reference tree are highlighted with arrowheads.
(0.67 MB TIF)

Figure S2 **Validation of generalist TF target promoters by ChIP-qPCR in** ***C. albicans*** **(A) and** ***S. cerevisiae*** **(B).** (C) Validation of the occupancy of Tbf1-TAP, Ifh1-TAP, and Fhl1-TAP at RP gene promoters and the rDNA control regions (NTS1 and NTS2) in *C. albicans* by ChIP-qPCR. Error bars reflect one standard deviation from the mean of three independent biological replicates.
(0.21 MB TIF)

Figure S3 **Validation of ChIP-CHIP thresholds.** (A) Distribution of signal intensities for each transcription factor and in each species. The threshold for each experiment (Z score of 2.0) is shown as a black bar. (B) The *p* value of overlap of orthologous TF regulons across species is threshold insensitive. Randomization with (C) and without (D) correction for promoter length shows that long promoters are an inherent source of experimental noise at Z score values below 1.5. The threshold used (2.0) is displayed as a dashed line.
(0.47 MB TIF)

Figure S4 **Enrichment of TF target gene sets for ribosome, carbon utilization, respiration, and sulfur/amino acid biosynthesis GO categories is robust to threshold.** The heatmap depicts the strength (log_10_ *p* value) of TF-GO interactions.
(0.33 MB TIF)

Figure S5 **Binding of Cbf1 to all** ***S. cerevisiae*** **centromeric regions.** No significant binding was observed for Hmo1, Rap1, Ifh1, Fhl1, and Tbf1.
(0.67 MB TIF)

Figure S6 **Results of ChIP-CHIP experiments showing binding to transcription factors (A and B) and central cell cycle regulators (C and D) gene promoters by generalist transcription factors in** ***S. cerevisiae*** **(B and D) and** ***C. albicans*** **(A and C).**
(0.88 MB TIF)

Figure S7 **Evolution of TF interactions with promoters of genes involved in cell signaling and the cell cycle.** A map of all significant regulatory relationships between each pleiotropic TF and kinases or cyclins listed in the kinase database (kinase.com; http://kinase.com/scerevisiae/yeastkinase.htm) was drawn.
(0.20 MB TIF)

Figure S8 **Evolution of the co-occurrence of generalist TFs in promoter regions within species.** (A) Heatmap reflecting the *p* value of the overlaps of target genes of generalist TFs within and between species in the subset of orthologous genes conserved between *S. cerevisiae* and *C. albicans*. Within species overlaps between the sets of targets of Cbf1 and Rap1 (A), Cbf1 and Tbf1 (B), and Rap1 and Tbf1 (C) in *C. albicans* and *S. cerevisiae*. The *p* values of each overlap were calculated using a hypergeometric distribution and are shown beneath each Venn diagram. NS stands for nonsignificant overlap.
(0.23 MB TIF)

Figure S9 **Validation of the Rap1 motifs obtained in** ***C. albicans*** **(A, C, and D) and** ***S. cerevisiae*** **(B).** The *C. albicans* Rap1 motif (A) is highly enriched at Rap1-bound regions (33/40 regions above a Z score of 2.0). The *S. cerevisiae* motif derived from our data is consistent across various randomized sets of 40 Rap1 target promoters (same size as the regulon of *C. albicans* Rap1) (C). The *C. albicans* consensus is clearly partitioned in two classes (C), one of which (class I) includes the *C. albicans* telomeric repeat (D; arrowhead).
(0.63 MB TIF)

Figure S10 **Phenotypic characterization of transcription-factor-conditional-mutants in** ***C. albicans*** **.** (A) The *ifh1/tetO-IFH1* and *fhl1/tetO-FHL1* conditional mutants are growth defective in rich medium. Ten-fold dilutions of the indicated strains were spotted on YPD with or without 100 µg/ml of tetracycline. (B) Effect of tetracycline shutoff of *CBF1*, *HMO1*, *IFH1*, *FHL1*, *TBF1*, and *TOR2* expression on rRNA abundance as observed on a total RNA electropherogram (RFU: Relative Fluorescence Units). (C) Expression profiling of ribosomal genes in conditional mutants shows that Ifh1, Fhl1, Tbf1, and Tor2 shutoff specifically down-regulate RP genes. RP genes are also down-regulated after rapamycin treatment and in a *cdc35Δ/cdc35Δ* mutant. Time of tetracycline or rapamycin treatment in hours is shown.
(1.82 MB TIF)

Figure S11 **Results of ChIP-CHIP experiments showing the enrichment profiles of various TFs at 10 randomly chosen RP genes of** ***C. albicans*** **(A) and** ***S. cerevisiae*** **(B).**
(1.03 MB TIF)

Figure S12 **Distribution of the pairwise distances (in bp) between peaks of enrichments of transcription factors occupying ribosomal protein promoters in** ***S. cerevisiae*** **(A) and** ***C. albicans*** **(B).**
(0.21 MB TIF)

Figure S13 **Ifh1 and Fhl1 interact in a nutrient- and stress-dependent fashion in** ***C. albicans*** **.** The yeast two-hybrid assay (Y2H) confirms that the Fhl1-Ifh1 heterotypic interaction occurs within and between species through their FHA and FHB domains, respectively. The FHA and FHB domains were expressed from pGADT7 and pGBKT7 Y2H vectors and monitored by growth on selective media (A) or beta-galactosidase assays (B). (C) Co-immunoprecipitation of full-length in vivo tagged Fhl1-HA and Ifh1-Myc after rapamycin treatment and various stresses (heat shock, osmotic shock, and hypoxia). (D) Model of the ribosomal protein regulatory complex of *C. albicans* and *S. cerevisiae*.
(0.51 MB TIF)

Table S1 **Strains used in this study.**
(0.17 MB DOC)

Text S1 **Supporting results.**
(0.11 MB DOC)

## Abbreviations

CGD: *Candida* genome database
ChIP: chromatin immunoprecipitation
GO: gene ontology
RP: ribosomal protein
SGD: *Saccharomyces* genome database
TF: transcription factor
TRN: transcriptional regulatory network
WCE: whole-cell extract
