# Supplementary figures and images for: Evolutionary Tinkering with Conserved Components of a Transcriptional Regulatory Network
Source: PLoS Biol. 2010 Mar 9;8(3):e1000329. doi: 10.1371/journal.pbio.1000329 (PMC2834713; doi:10.1371/journal.pbio.1000329)

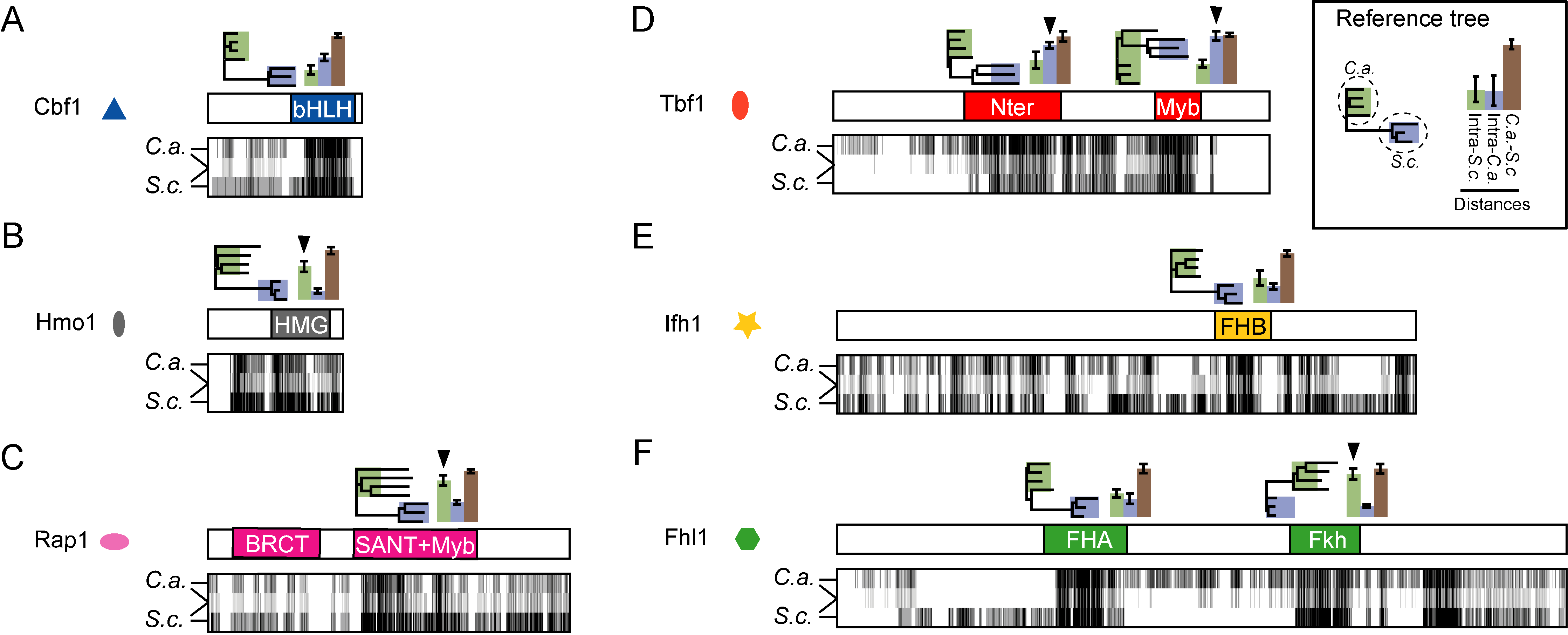

Supplement: Figure S1 — Variation in the primary sequence and domain organization of orthologous TFs. Alignment similarity maps of orthologs of the transcription factors (TFs) Cbf1, Hmo1, Rap1, Tbf1, Fhl1, and Ifh1 involved in the ribosomal protein (RP) transcriptional regulatory network of S. cerevisiae or C. albicans. C.a. and S.c. stand for C. albicans and S. cerevisiae and were used in all figures. Shading of the alignments reflects the percentage of conservation within the C. albicans (C. albicans, Pichia stipitis, Debaryomyces hansenii, and C. guilliermondii) or the S. cerevisiae (S. cerevisiae, Ashbya gossipii, and Kluvyeromyces lactis) branches or between the two branches. Histograms reflect the average phylogenetic distance derived from the PHYLIP distance matrix within (intra-S.c. and intra-C.a.) or between (C.a.-S.c.) branches. Distances showing a significant difference (p<0.01) compared to the reference tree are highlighted with arrowheads. (0.67 MB TIF) [file pbio.1000329.s003.tif]

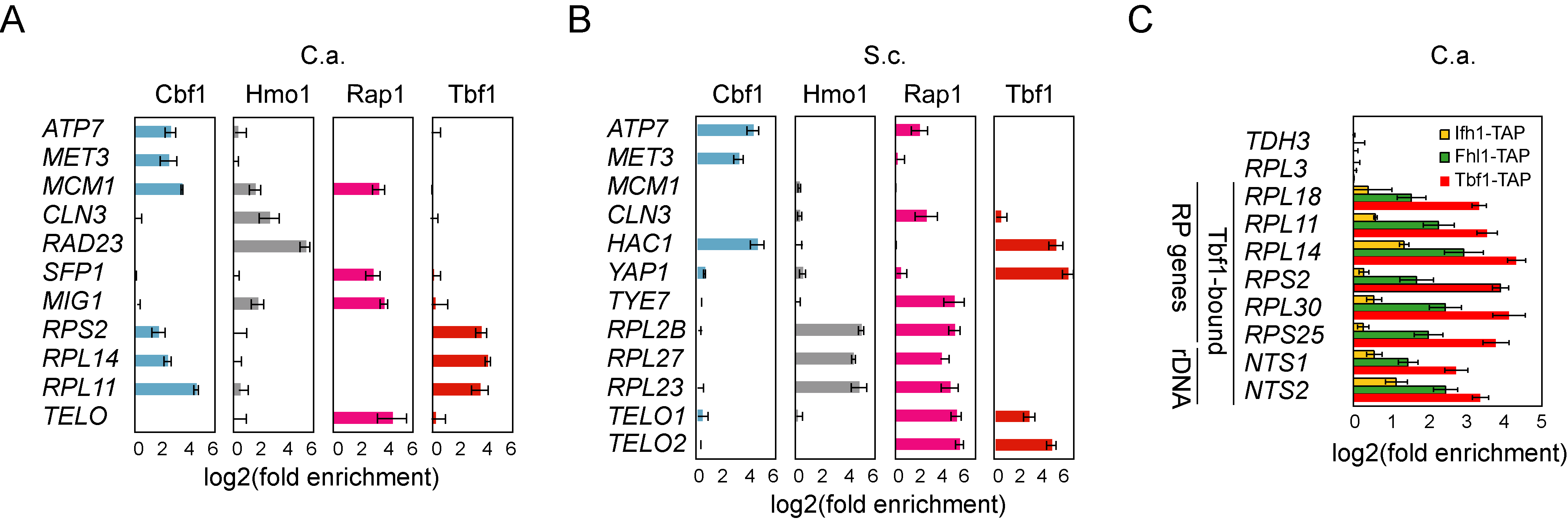

Supplement: Figure S2 — Validation of generalist TF target promoters by ChIP-qPCR in C. albicans (A) and S. cerevisiae (B). (C) Validation of the occupancy of Tbf1-TAP, Ifh1-TAP, and Fhl1-TAP at RP gene promoters and the rDNA control regions (NTS1 and NTS2) in C. albicans by ChIP-qPCR. Error bars reflect one standard deviation from the mean of three independent biological replicates. (0.21 MB TIF) [file pbio.1000329.s004.tif]

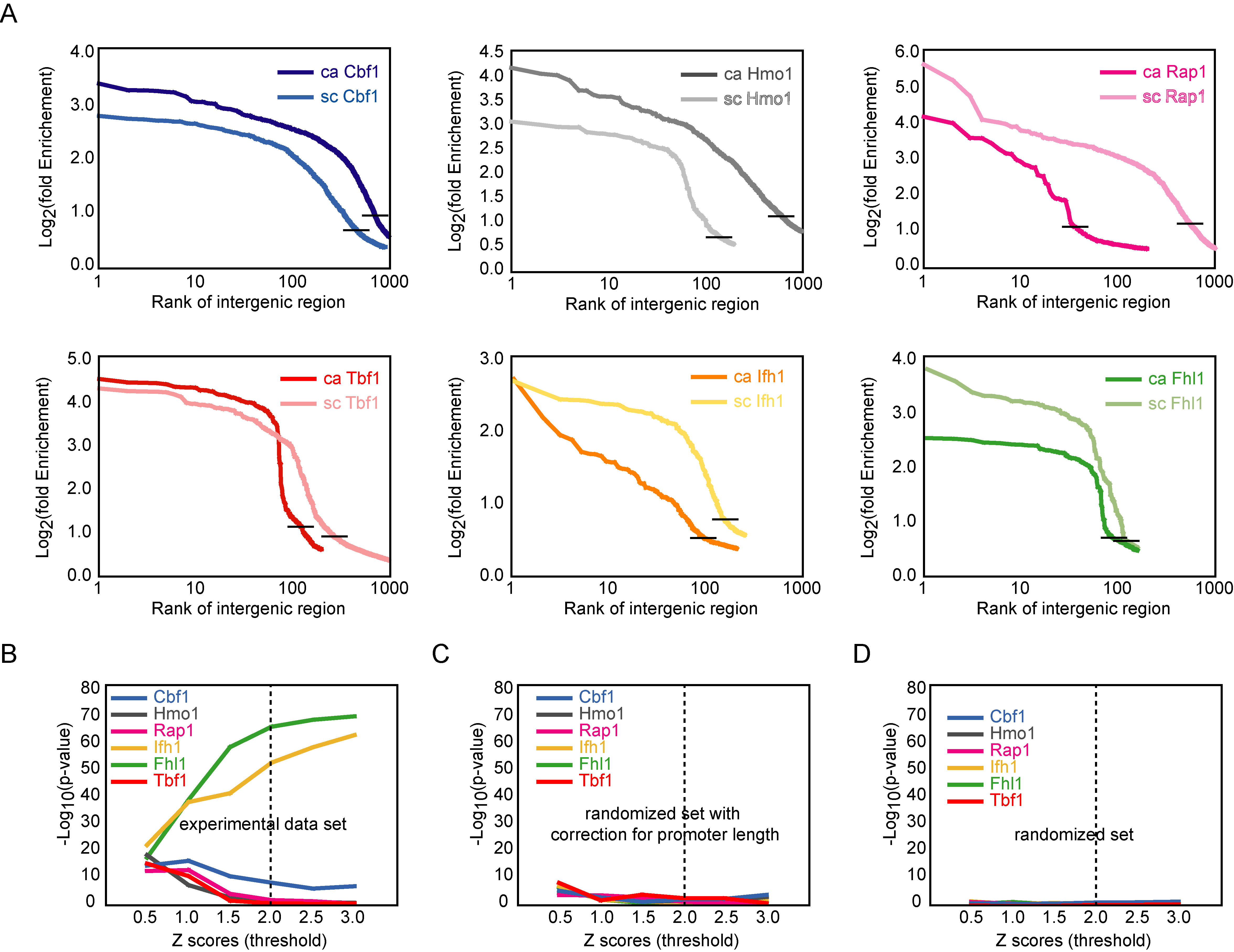

Supplement: Figure S3 — Validation of ChIP-CHIP thresholds. (A) Distribution of signal intensities for each transcription factor and in each species. The threshold for each experiment (Z score of 2.0) is shown as a black bar. (B) The p value of overlap of orthologous TF regulons across species is threshold insensitive. Randomization with (C) and without (D) correction for promoter length shows that long promoters are an inherent source of experimental noise at Z score values below 1.5. The threshold used (2.0) is displayed as a dashed line. (0.47 MB TIF) [file pbio.1000329.s005.tif]

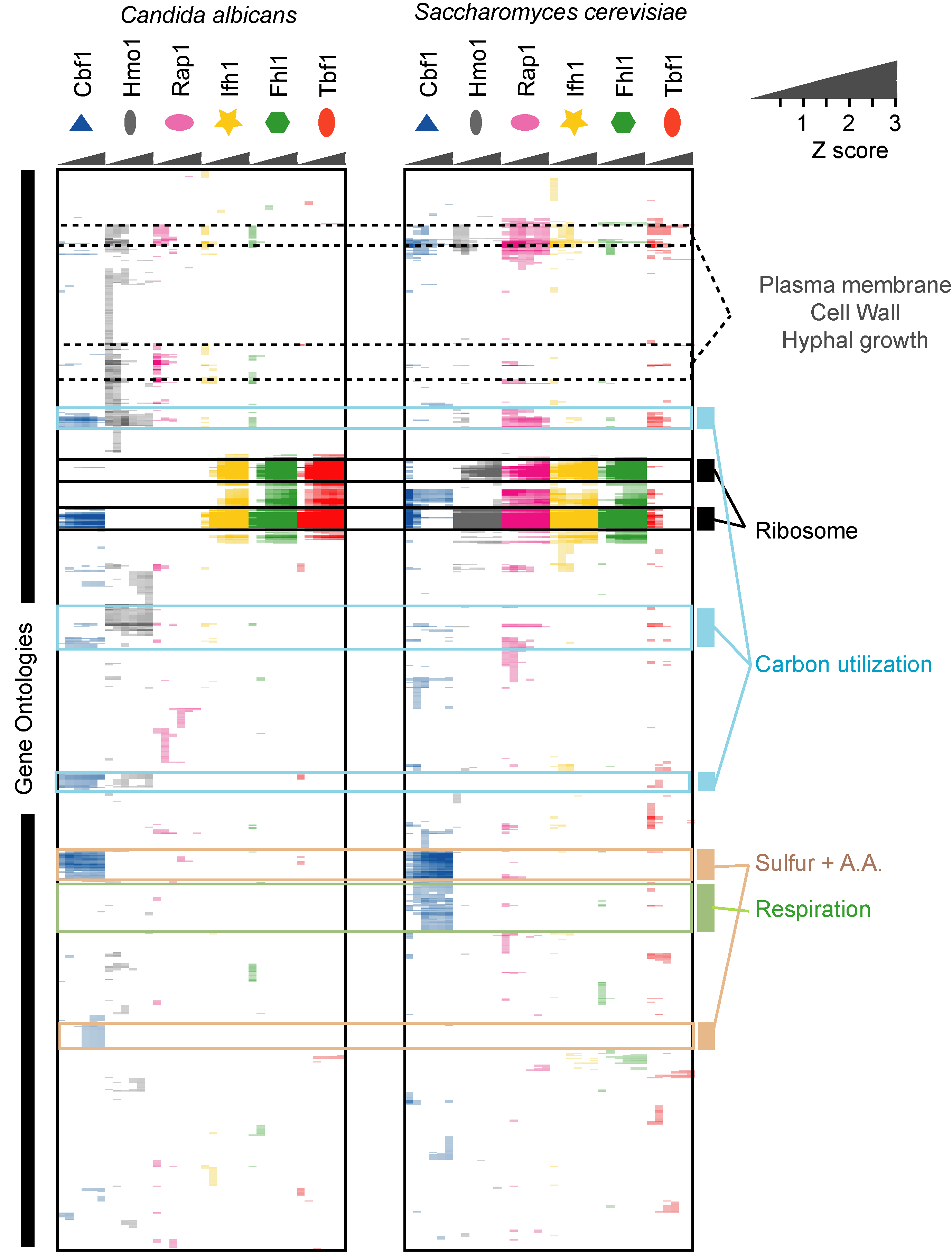

Supplement: Figure S4 — Enrichment of TF target gene sets for ribosome, carbon utilization, respiration, and sulfur/amino acid biosynthesis GO categories is robust to threshold. The heatmap depicts the strength (log10 p value) of TF-GO interactions. (0.33 MB TIF) [file pbio.1000329.s006.tif]

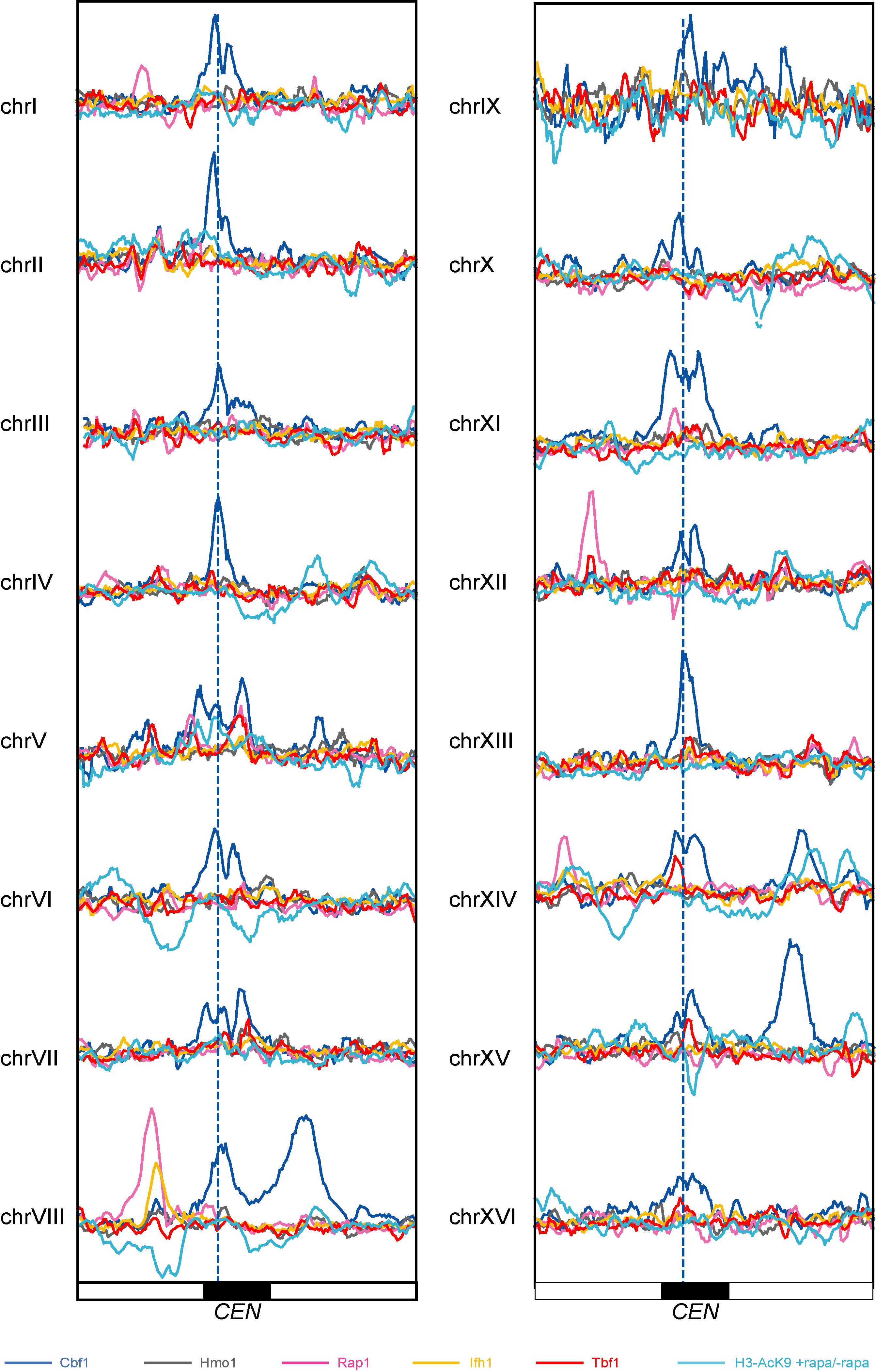

Supplement: Figure S5 — Binding of Cbf1 to all S. cerevisiae centromeric regions. No significant binding was observed for Hmo1, Rap1, Ifh1, Fhl1, and Tbf1. (0.67 MB TIF) [file pbio.1000329.s007.tif]

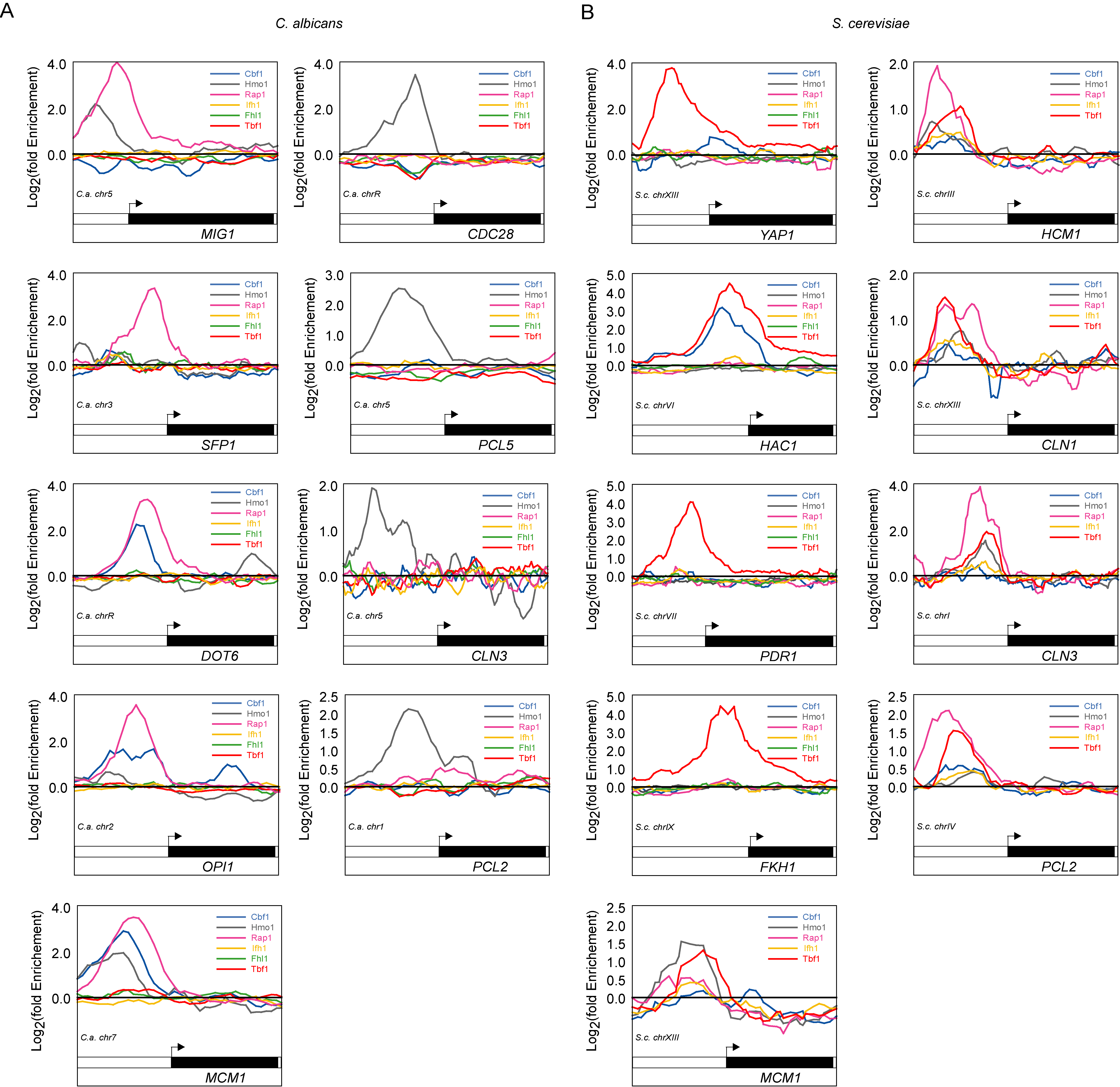

Supplement: Figure S6 — Results of ChIP-CHIP experiments showing binding to transcription factors (A and B) and central cell cycle regulators (C and D) gene promoters by generalist transcription factors in S. cerevisiae (B and D) and C. albicans (A and C). (0.88 MB TIF) [file pbio.1000329.s008.tif]

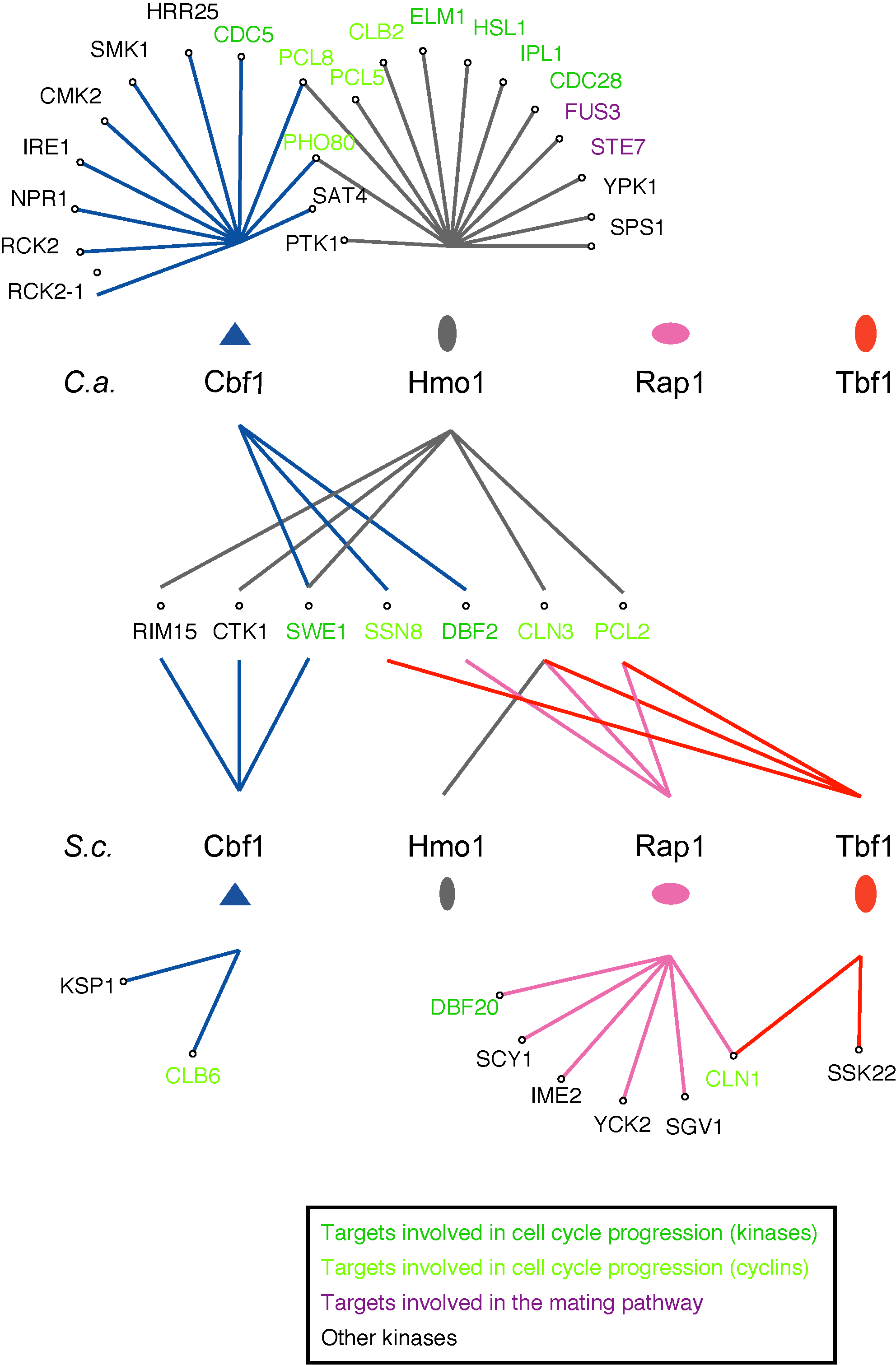

Supplement: Figure S7 — Evolution of TF interactions with promoters of genes involved in cell signaling and the cell cycle. A map of all significant regulatory relationships between each pleiotropic TF and kinases or cyclins listed in the kinase database (kinase.com; http://kinase.com/scerevisiae/yeastkinase.htm) was drawn. (0.20 MB TIF) [file pbio.1000329.s009.tif]

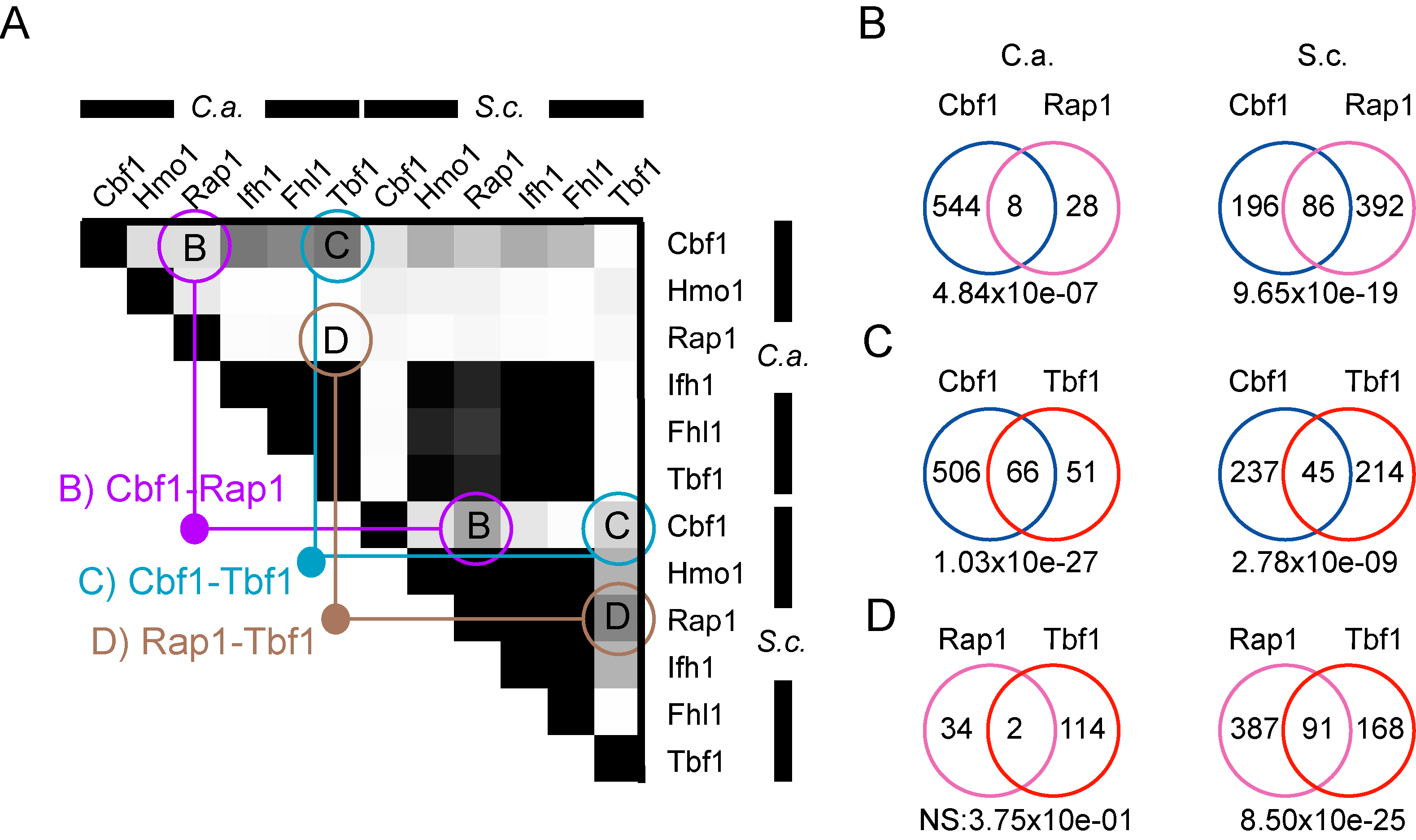

Supplement: Figure S8 — Evolution of the co-occurrence of generalist TFs in promoter regions within species. (A) Heatmap reflecting the p value of the overlaps of target genes of generalist TFs within and between species in the subset of orthologous genes conserved between S. cerevisiae and C. albicans. Within species overlaps between the sets of targets of Cbf1 and Rap1 (A), Cbf1 and Tbf1 (B), and Rap1 and Tbf1 (C) in C. albicans and S. cerevisiae. The p values of each overlap were calculated using a hypergeometric distribution and are shown beneath each Venn diagram. NS stands for nonsignificant overlap. (0.23 MB TIF) [file pbio.1000329.s010.tif]

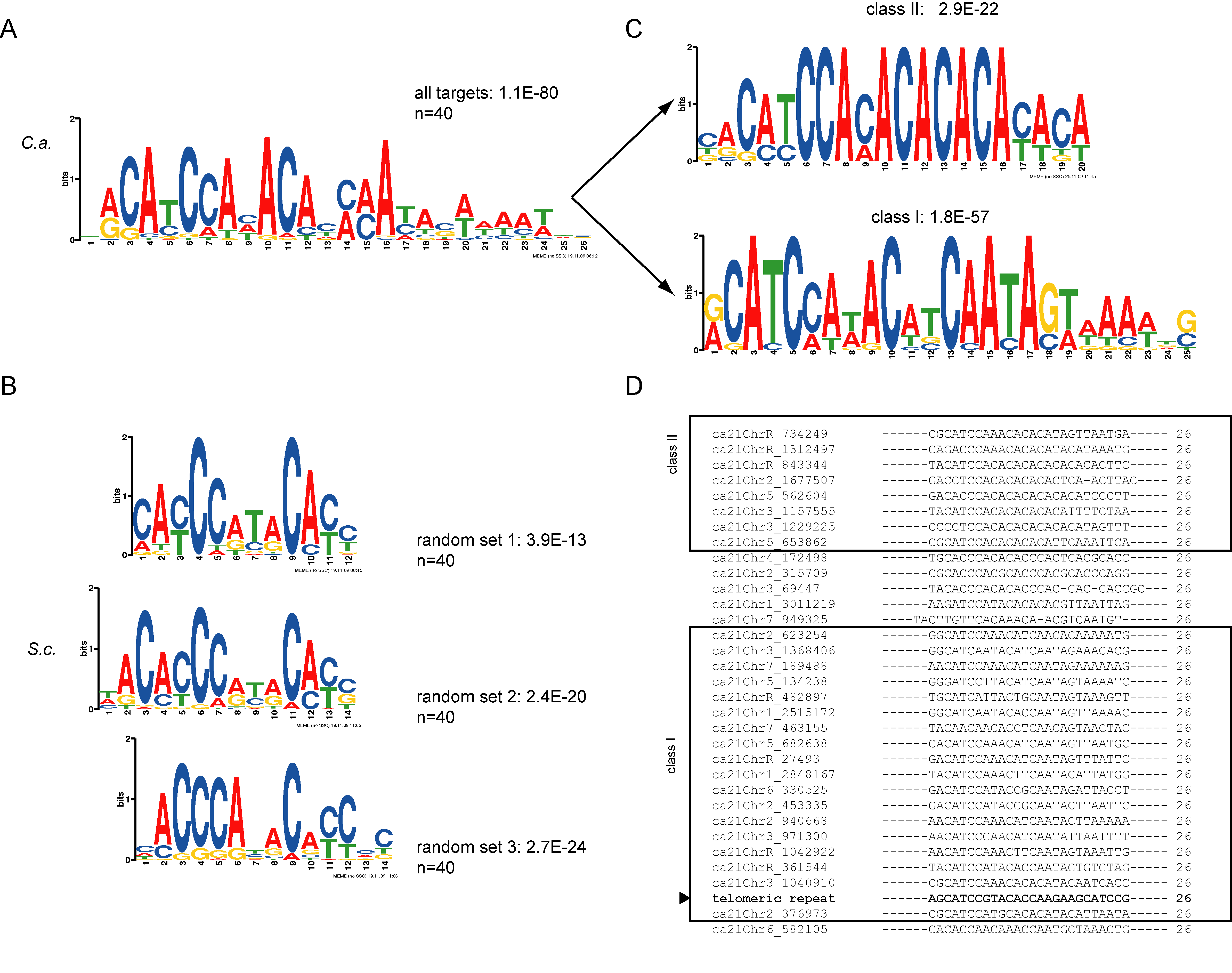

Supplement: Figure S9 — Validation of the Rap1 motifs obtained in C. albicans (A, C, and D) and S. cerevisiae (B). The C. albicans Rap1 motif (A) is highly enriched at Rap1-bound regions (33/40 regions above a Z score of 2.0). The S. cerevisiae motif derived from our data is consistent across various randomized sets of 40 Rap1 target promoters (same size as the regulon of C. albicans Rap1) (C). The C. albicans consensus is clearly partitioned in two classes (C), one of which (class I) includes the C. albicans telomeric repeat (D; arrowhead). (0.63 MB TIF) [file pbio.1000329.s011.tif]

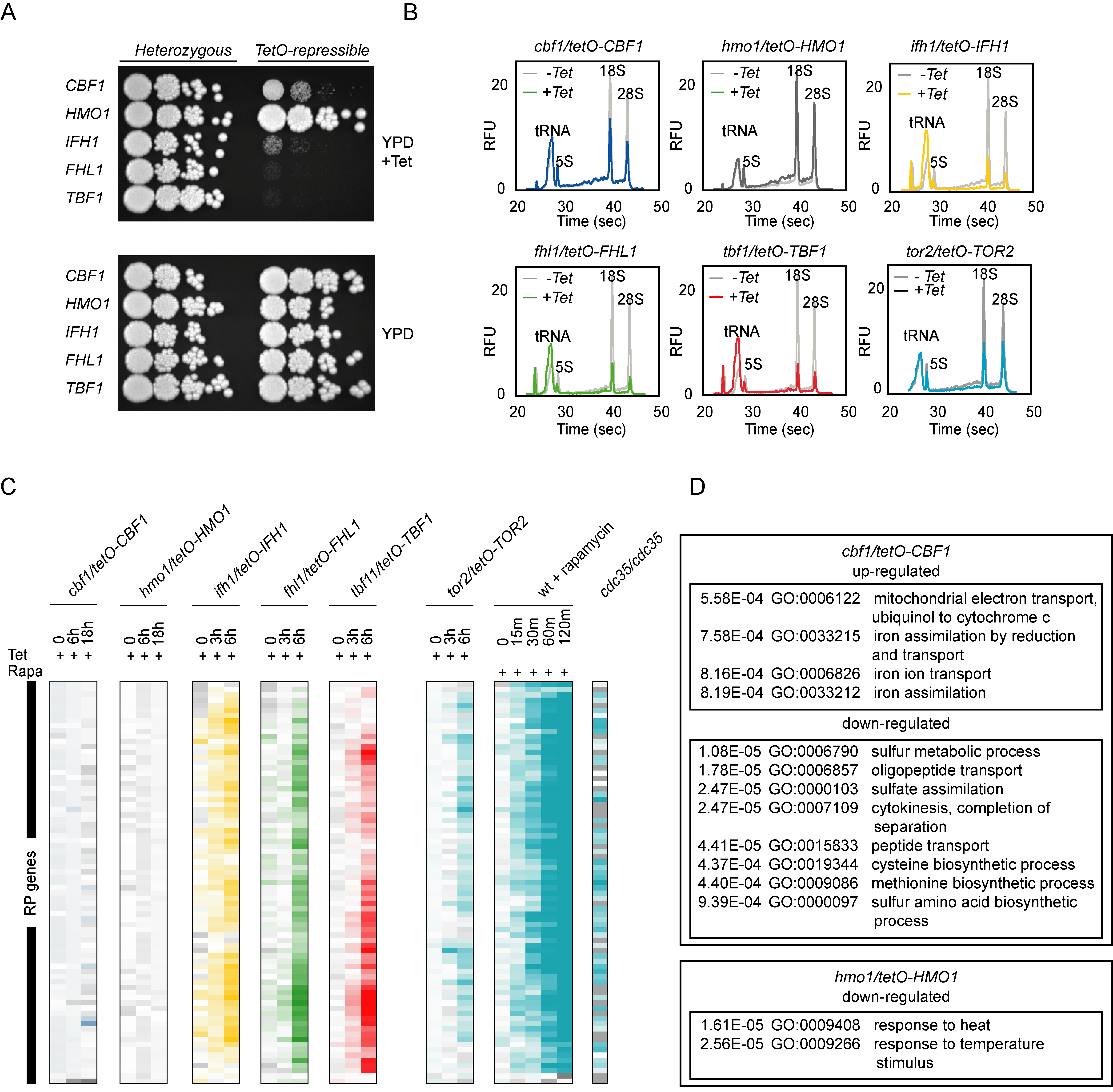

Supplement: Figure S10 — Phenotypic characterization of transcription-factor-conditional-mutants in C. albicans . (A) The ifh1/tetO-IFH1 and fhl1/tetO-FHL1 conditional mutants are growth defective in rich medium. Ten-fold dilutions of the indicated strains were spotted on YPD with or without 100 µg/ml of tetracycline. (B) Effect of tetracycline shutoff of CBF1, HMO1, IFH1, FHL1, TBF1, and TOR2 expression on rRNA abundance as observed on a total RNA electropherogram (RFU: Relative Fluorescence Units). (C) Expression profiling of ribosomal genes in conditional mutants shows that Ifh1, Fhl1, Tbf1, and Tor2 shutoff specifically down-regulate RP genes. RP genes are also down-regulated after rapamycin treatment and in a cdc35Δ/cdc35Δ mutant. Time of tetracycline or rapamycin treatment in hours is shown. (1.82 MB TIF) [file pbio.1000329.s012.tif]

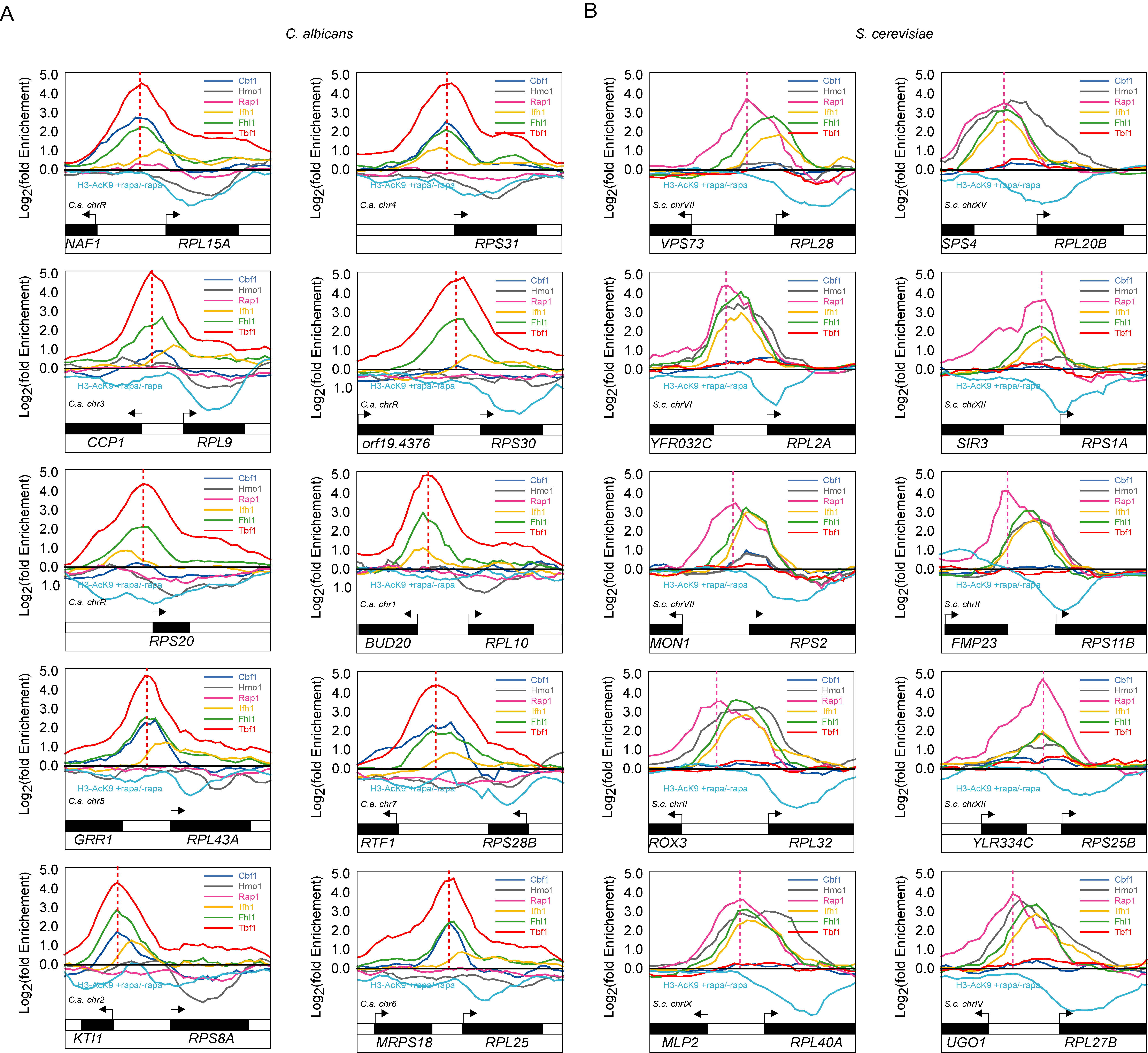

Supplement: Figure S11 — Results of ChIP-CHIP experiments showing the enrichment profiles of various TFs at 10 randomly chosen RP genes of C. albicans (A) and S. cerevisiae (B). (1.03 MB TIF) [file pbio.1000329.s013.tif]

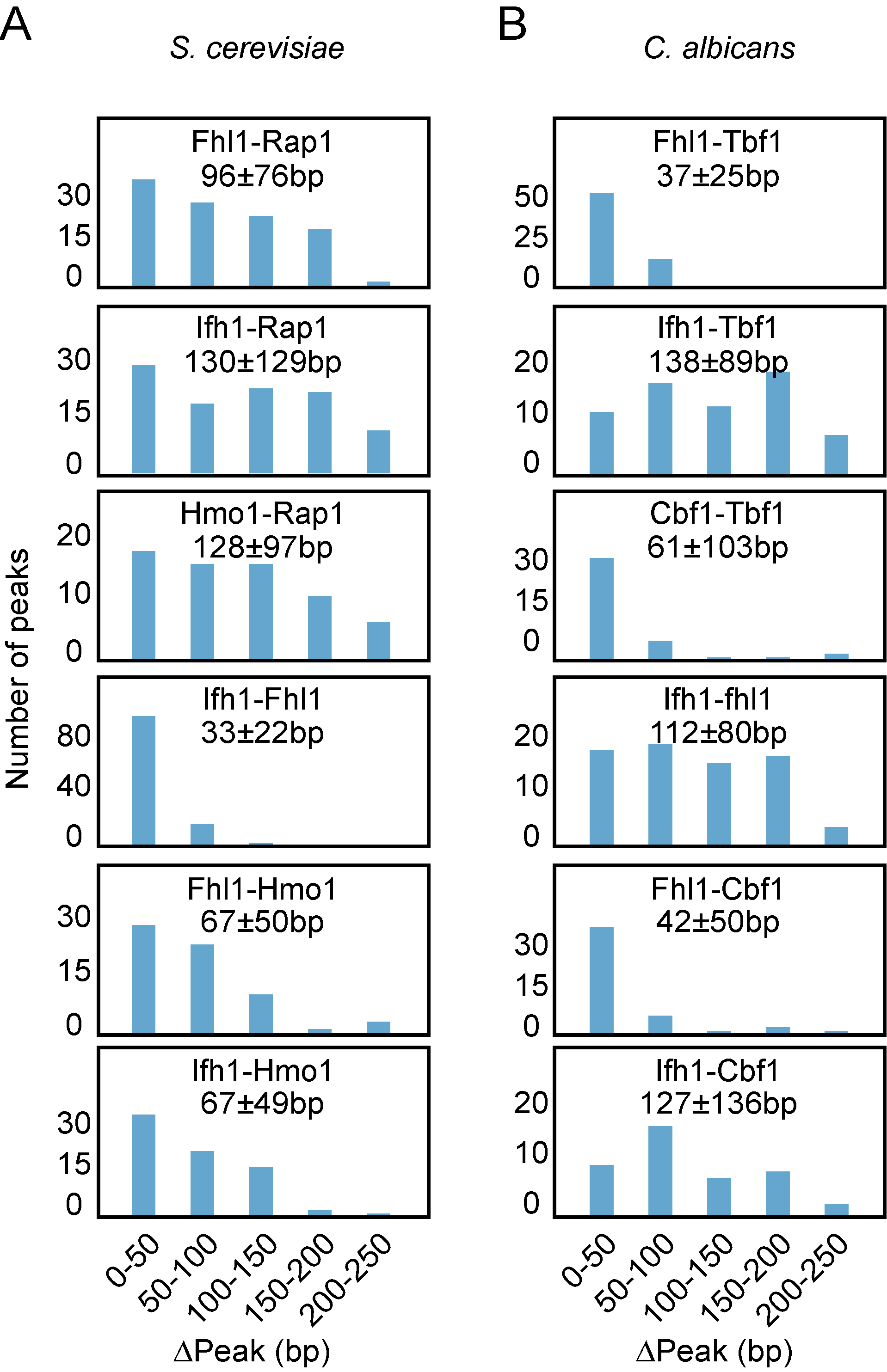

Supplement: Figure S12 — Distribution of the pairwise distances (in bp) between peaks of enrichments of transcription factors occupying ribosomal protein promoters in S. cerevisiae (A) and C. albicans (B). (0.21 MB TIF) [file pbio.1000329.s014.tif]

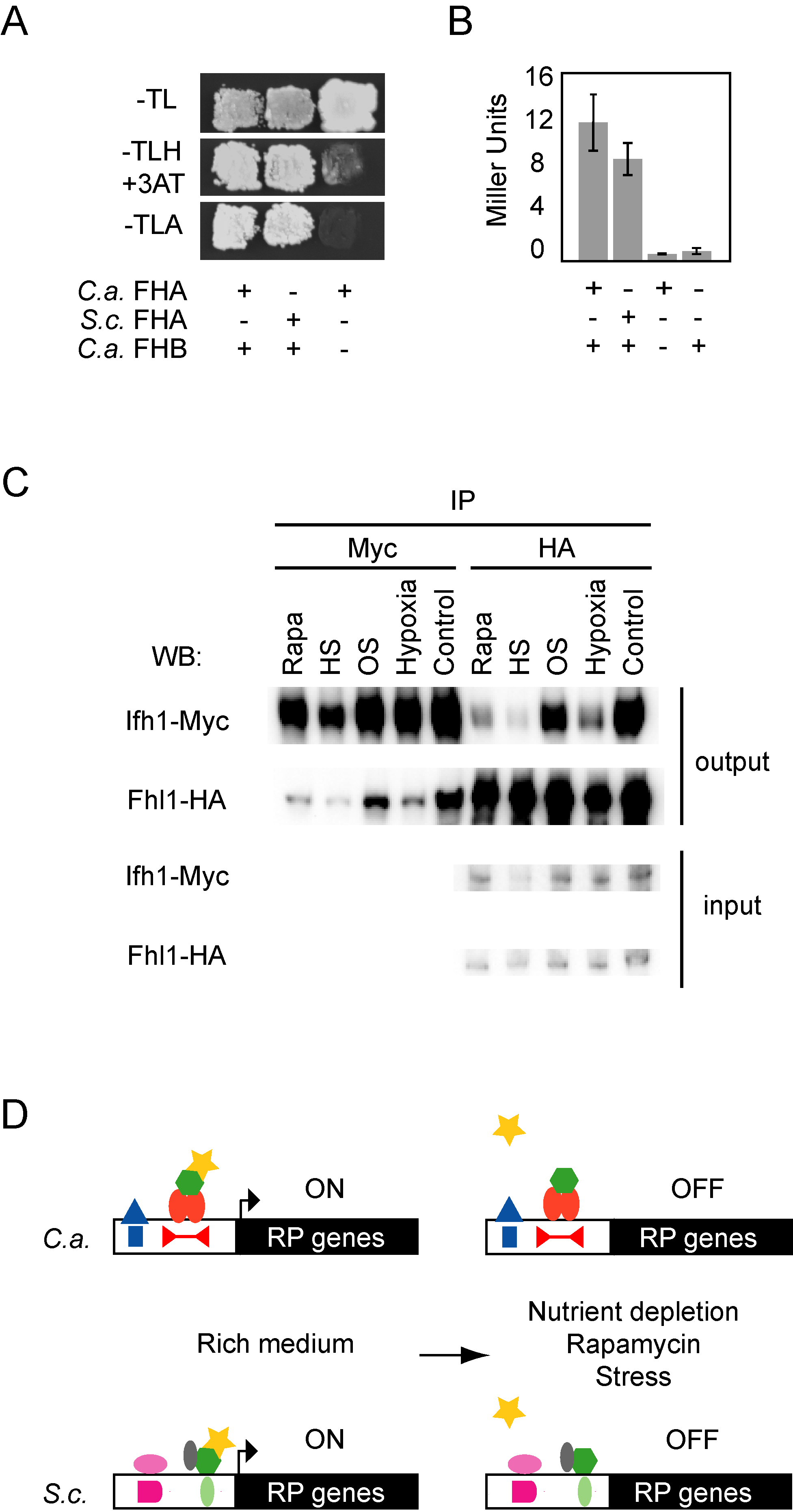

Supplement: Figure S13 — Ifh1 and Fhl1 interact in a nutrient- and stress-dependent fashion in C. albicans . The yeast two-hybrid assay (Y2H) confirms that the Fhl1-Ifh1 heterotypic interaction occurs within and between species through their FHA and FHB domains, respectively. The FHA and FHB domains were expressed from pGADT7 and pGBKT7 Y2H vectors and monitored by growth on selective media (A) or beta-galactosidase assays (B). (C) Co-immunoprecipitation of full-length in vivo tagged Fhl1-HA and Ifh1-Myc after rapamycin treatment and various stresses (heat shock, osmotic shock, and hypoxia). (D) Model of the ribosomal protein regulatory complex of C. albicans and S. cerevisiae. (0.51 MB TIF) [file pbio.1000329.s015.tif]
