# Supplementary material for: Evolutionary Tinkering with Conserved Components of a Transcriptional Regulatory Network
Source: PLoS Biol. 2010 Mar 9;8(3):e1000329. doi: 10.1371/journal.pbio.1000329 (PMC2834713; doi:10.1371/journal.pbio.1000329)
Supplement: Table S1 — Strains used in this study. (0.17 MB DOC) [file pbio.1000329.s016.doc]

**Table S1. Strains used in this study**

| **ID** | **Name** | **Genotype** | **Reference** |
| --- | --- | --- | --- |
| **MWY459** | BY4741 | MATa his3_1 leu2_0 met15_0 ura3_0 | [1] |
| **MWY486** | sc_Cbf1-TAP | BY4741 except for cbf1-TAP::HIS3MX6 | [2] |
| **MWY485** | sc_Hmo1-TAP | BY4741 except for hmo1-TAP::HIS3MX6 | [2] |
| **MWY483** | sc_Ifh1-TAP | BY4741 except for ifh1-TAP::HIS3MX6 | [2] |
| **MWY482** | sc_Tbf1-TAP | BY4741 except for tbf1-TAP::HIS3MX6 | [2] |
| **-** | W303 | ade2-1 leu2-3,112 ura3-1 his3-11,15 trp1-1 can1-100 ssd1-1 | [3] |
| **MWY487** | sc_TAP-Rap1 | W303 except for MATalpha TAP-rap1::TRP1 | [4] |
| **MWY500** | sc_Fhl1-HA | W303 except for MATa fhl1-HA3::G418r | [4] |
| **MWY501** | sc_Fhl1-HA Ifh1-Myc | W303 except for Mata ifh1-Myc9-Trp1+, fhl1-3HA-G418 | [4] |
| **MWC695** | BWP17 | ura3::imm434/ura3::imm434 his1::hisG/his1::hisG arg4::hisG arg4::hisG | [5] |
| **MWC713** | ca_Cbf1-TAP | BWP17 except for cbf1-TAP::URA3 | [6,7] |
| **MWC855** | ca_Hmo1-TAP | BWP17 except for hmo1-TAP::URA3 | This study |
| **MWC718** | ca_Rap1-TAP | BWP17 except for rap1-TAP::URA3 | This study |
| **MWC719** | ca_Ifh1-TAP | BWP17 except for ifh1-TAP::URA3 | This study |
| **MWC720** | ca_Fhl1-TAP | BWP17 except for fhl1-TAP::URA3 | This study |
| **MWC715** | ca_Tbf1-TAP | BWP17 except for tbf1-TAP::URA3 | [6,7] |
| **MWC726** | CaSS1 | his3::hisG/his3::hisG leu2::tetR-GAL4AD-URA3/LEU2 | [8] |
| **MWC739** | ca_Cbf1-tetR | CaSS1 except for cbf1::HIS3/pTet-CBF1 | [8] |
| **MWC740** | ca_Cbf1-hetero | CaSS1 except for CBF1/cbf1::HIS3 | [8] |
| **MWC717** | ca_Tbf1-tetR | CaSS1 except for tbf1::HIS3/pTet-TBF1 | [8] |
| **MWC708** | ca_Tbf1-hetero | CaSS1 except for TBF1/tbf1::HIS3 | [8] |
| **MWC732** | ca_Ifh1-tetR | CaSS1 except for ifh1::HIS3/pTet-IFH1 | [8] |
| **MWC731** | ca_Ifh1-hetero | CaSS1 except for IFH1/ifh1::HIS3 | [8] |
| **MWC727** | ca_Fhl1-tetR | CaSS1 except for fhl1::HIS3/pTet-FHL1 | This study |
| **MWC723** | ca_Fhl1-hetero | CaSS1 except for FHL1/fhl1::HIS3 | This study |
| **MWC858** | ca_Hmo1-tetR | CaSS1 except for hmo1::HIS3/pTet-HMO1 | This study |
| **MWC857** | ca_Hmo1-hetero | CaSS1 except for HMO1/hmo1::HIS3 | This study |

**References**

1. Brachmann CB, Davies A, Cost GJ, Caputo E, Li J, et al. (1998) Designer deletion strains derived from Saccharomyces cerevisiae S288C: a useful set of strains and plasmids for PCR-mediated gene disruption and other applications. Yeast 14: 115-132.

2. Ghaemmaghami S, Huh WK, Bower K, Howson RW, Belle A, et al. (2003) Global analysis of protein expression in yeast. Nature 425: 737-741.

3. Thomas BJ, Rothstein R (1989) Elevated recombination rates in transcriptionally active DNA. Cell 56: 619-630.

4. Rudra D, Zhao Y, Warner JR (2005) Central role of Ifh1p-Fhl1p interaction in the synthesis of yeast ribosomal proteins. Embo J 24: 533-542.

5. Wilson RB, Davis D, Mitchell AP (1999) Rapid hypothesis testing with Candida albicans through gene disruption with short homology regions. J Bacteriol 181: 1868-1874.

6. Lavoie H, Sellam A, Askew C, Nantel A, Whiteway M (2008) A toolbox for epitope-tagging and genome-wide location analysis in Candida albicans. BMC Genomics 9: 578.

7. Hogues H, Lavoie H, Sellam A, Mangos M, Roemer T, et al. (2008) Transcription factor substitution during the evolution of fungal ribosome regulation. Mol Cell 29: 552-562.

8. Roemer T, Jiang B, Davison J, Ketela T, Veillette K, et al. (2003) Large-scale essential gene identification in Candida albicans and applications to antifungal drug discovery. Mol Microbiol 50: 167-181.
