# Supplementary material for: Evolutionary Tinkering with Conserved Components of a Transcriptional Regulatory Network
Source: PLoS Biol. 2010 Mar 9;8(3):e1000329. doi: 10.1371/journal.pbio.1000329 (PMC2834713; doi:10.1371/journal.pbio.1000329)
Supplement: Text S1 — Supporting results. (0.11 MB DOC) [file pbio.1000329.s017.doc]

**Supplementary on-line information for:**

# ­Evolutionary tinkering with conserved components of a transcriptional regulatory network

Hugo Lavoie^1,2,4^, Hervé Hogues^1^, Jaideep Mallick^1^, Adnane Sellam^1,3^, André Nantel^1,3^ and Malcolm Whiteway^1,2^

^1^Biotechnology Research Institute, National Research Council, Montreal, Quebec, H4P 2R2, Canada

^2^ Department of Biology, McGill University, Montreal, Quebec, H3A 1B1, Canada

^3^Department of Anatomy and Cell Biology, McGill University, Montreal, Quebec, H3A 1B1, Canada

^4^Current address: Intracellular Signaling Laboratory, Institute of Research in Immunology and Cancer (IRIC), University of Montreal, Montreal, Quebec H3C 3J7, Canada.

**Supplementary results**

**Resequencing of the Ifh1 gene**

A careful analysis of the *C. albicans* Ifh1 locus (orf19.4281) revealed that it encoded only the C.-terminal portion of *S. cerevisiae* Ifh1. After blasting the *S. cerevisiae* Ifh1 protein against all *C. albicans* orf19 translations, we found that orf19.4281 neighboring orf19.4282 encoded a conserved N-terminal portion of *S. cerevisiae* Ifh1. In addition, *Candida tropicalis, Pichia stipitis, Debaryomyces* *hansenii* and *Candida guilliermondii* all have a single ORF spanning orf19.4281 and orf19.4282. We inspected sequencing data from the *C. albicans* genome-sequencing project (http://candida.bri.nrc.ca/candida/index.cfm) and realized that low quality reads clustered between the two ORFs. Resequencing of this region revealed that orf19.4281 and orf19.4282 form in fact a single uninterrupted translational unit of 907 amino acids.

**Protein sequence evolution of orthologous TFs**

The DNA-binding and transactivating domains of TFs very often act as independent functional units and therefore evolve mostly independently at the sequence level [1,2]. Protein sequence evolution of the domains of TFs can thus be indicative of the evolution of their different functions [3,4]. We produced protein sequence alignments to visually display the level of sequence similarity amongst four species closely related to *C. albicans* (*C. albicans, Pichia stipitis, Debaryomyces* *hansenii* and *C. guilliermondii*) and three species of the *S. cerevisiae* branch (*S. cerevisiae, Ashbya gossipii* and *Kluvyeromyces lactis*) and compared similarities between the two clades for all six TFs. We also established neighbor-joining phylogenic trees of the conserved regions of all six proteins in addition to a reference tree drawn from highly conserved protein coding and RNA-coding sequences [5](Fig. S1). The Cbf1-bHLH, Hmo1-HMG, Rap1-Myb, Rap1-BRCT, Tbf1-Myb, Tbf1-N-terminal, Fhl1-FHA and Ifh1-FHB domains are very well conserved between species and could be used to derive phylogenies. Despite the presence of these conserved domains, all proteins have branch specific insertions or deletions, Fhl1 and Ifh1 being the most drastic examples with highly variable regions, insertions and deletions accounting for 57% and 43% of their sequence alignments respectively (Fig. S1).

Apart from the phylogenetic distances between the Cbf1-bHLH, Fhl1-FHA and Ifh1-FHB (Fig. S1A, E and F) domains, all protein regions appear to have experienced significant branch-specific rates of protein sequence variation (Intra-*S.c.* and Intra-*C.a.*; arrowheads) compared to the relative distances derived from the reference tree (Fig. S1). It is also noticeable that the substitution rates of individual domains can vary independently within the same TF. For example, this is supported by the length of the branches of phylogenetic trees derived individually for the FHA and Fkh domains of the Fhl1 protein.

Although Hmo1 has a highly conserved HMG-box domain, its protein sequence has a high divergence rate in the *C. albicans* lineage while it has remained mostly fixed in the *S. cerevisiae* lineage (Fig. S1B).

The Rap1 bipartite SANT-Myb domain has dramatically changed in the *C. albicans* lineage while it is highly conserved in the *S. cerevisiae* lineage. The N-terminal Domain 1 portion of the published crystal structure is equally conserved in both groups and the C-terminal Domain 2 has been subjected to extensive sequence variation in the *C. albicans* lineage [6]. The Rap1 protein is composed of two independent units arranged in a flexible tandem. The Myb Domain 1 of Rap1 is mostly responsible for the contacts with the conserved CACCC moiety of the Rap1 element and is highly conserved between the *S. cerevisiae* and *C. albicans* clades. In contrast, Domain 2 primary sequence contains conserved residues directly contacting the ACA moiety whereas the residues of Domain 2 contacting the 3’-most bases in the crystal structure have largely been substituted between species [6](Fig. S1). This is consistent with previous observations [7,8]. Otherwise, the BRCT domain found in *S. cerevisiae* Rap1 is hardly detectable (rpsBLAST) or alignable in any of the four species related to *C. albicans* (Fig. S1C).

The Fhl1 Forkhead domain has an accelerated amino acid substitution rate in the *C. albicans* lineage while it is stable in the *S. cerevisiae* group (Fig. S1F). In contrast, the Fhl1-FHA domain has followed the same rate of substitution in both lineages and has similar branch lengths as the reference tree. This trend is even more striking when the *S. cerevisiae* or *C. albicans* Fhl1 sequence is compared to its *Schizosaccharomyces pombe* paralog: in *S. pombe*, the FHA domain is strongly conserved while the Fkh sequence has been highly substituted. This suggests that the Fkh region, likely responsible for Fhl1 contact with DNA, is subjected to less selective pressure than the FHA domain.

Finally, Tbf1 comprises both a well-conserved Myb DNA-binding domain and a conserved N-terminal region of unknown function. The N-terminal region of Tbf1 has rapidly diverged in species related to *S. cerevisiae* but not in the *C. albicans* branch. Similarly, the Myb domain of Tbf1 has experienced accelerated sequence changes in the *S. cerevisiae* branch but is highly conserved in the species related to *C. albicans* (Fig. S1D).

Altogether, analysis of the primary sequence of conserved regions of the six TFs under study suggests that positive selection was applied on protein domains in the evolutionary time separating *S. cerevisiae* and *C. albicans*.

**Validation of thresholds used for ChIP-CHIP analysis**

The definition of thresholds is important when studying the evolution of transcription networks across species. Therefore, we established our ChIP-CHIP thresholds in a non-biased manner based on the distribution of signal intensities in our normalized data. Bound regions were defined as having a peak intensity at least two standard deviations above the mean of signal intensities (Z score of 2.0). The distribution of our ChIP-CHIP signal ratios display a common signature for all transcription factors in the two species with a plateau followed by a sharp decrease in signal strength in the enrichment vs rank plots (Fig. S3A). Our threshold values based on a Z score of 2.0 systematically fell at the bottom of this sharp slope (Fig. S3A; black line) and are consistent across species for all TFs; this suggests that our sets of target genes include most true targets and are above experimental noise. These unbiased thresholds gave targets lists compatible with previously published data for all *S. cerevisiae* TFs studied here [9-14].

Because of our very stringent threshold, the concern that we might misestimate regulon conservation of TFs and functional relationships between TFs and gene ontology (GO) categories arose during the course of our analysis. To address this possibility, we made a methodical analysis of our data by varying the threshold values from 0.5 to 3.0 Z scores (a threshold of 2.0 Z scores was used in the manuscript). The sets of target genes obtained for each TF and with each Z score were subjected to the analysis of regulon conservation across species of orthologous TFs (with the hypergeometric distribution in the space of *C. albicans-S. cerevisiae* orthologs; Figure S3B-D) and to GO enrichment analysis (Figure S4).

We observed that Z-scores between 1.5 and 3.0 yielded similar conservation of TF regulons and that Z scores of 0.5 and 1.0 gave a similar level of regulon overlap for all TFs (Fig. S3B). Similarly, we observed a lot of isolated GO enrichments after hierarchical clustering at low Z score values while Z-scores of 1.5 and above gave stable enrichment *p* values (Fig. S4). In additions, we observed that a few clusters of GO categories were enriched in many if not all TFs at low Z score values (Fig. S4; dashed box). This suggests that certain GO categories systematically generate experimental noise in the ChIP-CHIP procedure. Interestingly, most of these “noisy” functional associations were given for GO categories associated with the plasma membrane and cell wall compartments including adhesins and GPI-anchored proteins and involved in morphogenetic processes like hyphal growth adhesion. Interestingly, it was reported that these classes of genes harbor unusually long promoters in *C. albicans* and the budding yeast [15,16]. Our observations likely correlate with the fact that these large intergenic regions are more prone to generate noise across species. To test whether these long promoters account for part of the experimental noise, we randomly assigned our ChIP-CHIP signals to ORFs of *S. cerevisiae* and *C. albicans* in a manner either corrected (Fig. S3C) or uncorrected (Fig. S3D) for promoter lengths. We observed that correcting for promoter length in the randomization procedure caused an increase in target overlap between species for all TFs at low Z scores (Fig. S3C) while the fully randomized set gave no enrichment at any Z score value (Fig. S3D). We conclude from this that long promoters are an inherent source of noise in the study of TF binding across species and that any enrichment in these functions should be taken with caution.

For the simplified analysis presented in the middle panel of Figure 3A, only GO enrichment blocks (observed at any threshold for a given TF; ribosome, carbon utilization, respiration and sulfur starvation; Fig. S4) were taken into account. Altogether, it is apparent that our conclusion of dramatic qualitative changes in the regulons and functional connectivities of orthologous TFs is highly robust to threshold.

**Evaluation of TF-TF relationships of generalist regulators on promoter regions**

Since Hmo1 and Rap1 in *S. cerevisiae* and Cbf1 and Tbf1 in *C. albicans* clearly have common target promoters, we decided to examine more closely and systematically the evolution of intra-species interactions between generalist TFs. For this, we calculated the *p* value of the overlaps obtained by pairwise comparisons of the groups of target genes of each TF both inter and intra-species (Fig. S8). We excluded from this analysis all overlaps between regulators caused by their co-occurrence at RP genes since they are treated in the section specifically discussing the RP regulatory complex.

Rap1 and Cbf1 in *S. cerevisiae* contact a common set of 73 genes (58 in the *S. cerevisiae*-*C. albicans* orthologous space; Fig. S8B). Of these, 12 are RP (6 genes) and glycolysis genes (6 genes) and the remainder are genes with no GO category enrichment. This relationship appears conserved in *C. albicans* as the Rap1 and Cbf1 profiles overlap on the promoters of the TFs *MCM1* (Fig. S6 and S8), *OPI1*, *SEF1*, *DOT6*, *MET14* and *OP4*.

We previously showed that *C. albicans* Tbf1 and Cbf1 co-occur on RP gene promoters and that their respective elements are found in close proximity; this work also established that they likely bind to intergenic regions in a cooperative fashion [17]. We recapitulate this finding here with a highly significant overlap between the Cbf1 and Tbf1 target genes (*p*=7.26x10^-35^)(Fig. S8C). The overlap between these two TFs in *S. cerevisiae* also seems significant but no functional enrichment is observed. Finally, Rap1 and Tbf1 also overlap on a marginally significant proportion of their targets in *S. cerevisiae* but not in *C. albicans* (Fig. S8D). Of these targets, 6 are RP genes, 4 are glycolysis genes and 4 are cell cycle regulators.

**Expression profiling following rapamycin treatment and phenotypic and expression profiling of ribosomal regulators in *C. albicans***

First, we wanted to evaluate the phenotypic consequence of ablating the RP regulators of *C. albicans*. Since central ribosomal regulators are likely to be essential genes, we derived tetracycline repressible versions in *C. albicans* and monitored the cellular growth phenotype and expression profiles by microarray analysis. The Cbf1, Ifh1, Fhl1 and Tbf1 conditional mutants all display a severe growth phenotype (Fig. S10A). In contrast, the Hmo1 conditional mutant displays no visible phenotype even though the Hmo1 mRNA is depleted by 515±49 fold as tested by RT-qPCR (Fig. S10A). Similarly, the complete ablation of *RAP1* causes a slight decrease in growth rate confirming that *RAP1* is not essential in *C. albicans* although a *rap1Δ/rap1Δ* deletion strain exhibits a prominent telomere phenotype consistent with our findings that Rap1 is bound to telomeric repeats in *C. albicans* [8].

Since we previously observed that *TBF1* shutoff caused a marked decrease in rRNA abundance, we tested whether this phenotype was also present in other conditional mutants. The Fhl1 and Ifh1 shutoff strains basically phenocopy the *TBF1* mutant in that respect while *HMO1* depletion has no effect on rRNA abundance and the Cbf1 mutant is intermediate (Fig. S10B).

The expression profile of the Cbf1 conditional mutant after tetracycline treatment showed an important decrease in the expression of sulfur starvation genes and an increase in most genes of the respiratory regulon but no detectable change in the expression of RP subunits was detectable (Fig. S10C and D). *HMO1* shutoff caused no observable change in the expression profile of polyadenylated RNAs except a highly significant decrease in its own expression (Fig. S10C and D). Finally, transcriptional profiling of the Ifh1 and Fhl1 tetracycline-repressible mutants shows specific down-regulation of RP genes after tetracycline treatment as previously demonstrated with a Tbf1 conditional allele (Fig. S10C)[17].

Next, we asked whether the inhibition of signal transduction pathways known to affect *S. cerevisiae* RP genes transcription also affect the *C. albicans* ribosomal regulon. For this, since no expression profiles after TOR inhibition were available for *C. albicans* before February 2009 [18], we monitored gene expression with microarrays in a time course experiment following rapamycin treatment (1ng/ml of rapamycin was added to YPD). Consistently and as observed in *S. cerevisiae*, a reduction in PKA (in a *cdc35Δ/cdc35Δ* mutant) or TOR activity (rapamycin treatment or a tetracycline-repressible conditional mutant of *TOR2*) causes the most dramatic and systematic decrease in RP genes transcription (Fig. S10C) [19]. As well, as reported in *S. cerevisiae*, rapamycin treatment of *C. albicans* caused down-regulation of the ribosome biogenesis (Ribi) regulon involved in rRNA processing (data not show and) [18,20,21]. In addition, tetracycline shutoff of *TOR2* caused an important decrease in rRNA abundance (Fig. S10B) similar to what was previously seen in *S. cerevisiae* after TOR inhibition by rapamycin [22,23].

**References**

1. Reece RJ, Platt A (1997) Signaling activation and repression of RNA polymerase II transcription in yeast. Bioessays 19: 1001-1010.

2. Martchenko M, Levitin A, Whiteway M (2007) Transcriptional activation domains of the Candida albicans Gcn4p and Gal4p homologs. Eukaryot Cell 6: 291-301.

3. Lynch VJ, Wagner GP (2008) Resurrecting the role of transcription factor change in developmental evolution. Evolution 62: 2131-2154.

4. Wagner GP, Pyle AM (2007) Tinkering with transcription factor proteins: the role of transcription factor adaptation in developmental evolution. Novartis Found Symp 284: 116-125; discussion 125-119, 158-163.

5. James TY, Kauff F, Schoch CL, Matheny PB, Hofstetter V, et al. (2006) Reconstructing the early evolution of Fungi using a six-gene phylogeny. Nature 443: 818-822.

6. Konig P, Giraldo R, Chapman L, Rhodes D (1996) The crystal structure of the DNA-binding domain of yeast RAP1 in complex with telomeric DNA. Cell 85: 125-136.

7. Lue NF (2009) Plasticity of telomere maintenance mechanisms in yeast. Trends Biochem Sci.

8. Yu EY, Yen WF, Steinberg-Neifach O, Lue NF (2009) Rap1 in Candida albicans: an unusual structural organization and a critical function in suppressing telomere recombination. Mol Cell Biol.

9. Lee TI, Rinaldi NJ, Robert F, Odom DT, Bar-Joseph Z, et al. (2002) Transcriptional regulatory networks in Saccharomyces cerevisiae. Science 298: 799-804.

10. Harbison CT, Gordon DB, Lee TI, Rinaldi NJ, Macisaac KD, et al. (2004) Transcriptional regulatory code of a eukaryotic genome. Nature 431: 99-104.

11. Schawalder SB, Kabani M, Howald I, Choudhury U, Werner M, et al. (2004) Growth-regulated recruitment of the essential yeast ribosomal protein gene activator Ifh1. Nature 432: 1058-1061.

12. Wade JT, Hall DB, Struhl K (2004) The transcription factor Ifh1 is a key regulator of yeast ribosomal protein genes. Nature 432: 1054-1058.

13. Hall DB, Wade JT, Struhl K (2006) An HMG protein, Hmo1, associates with promoters of many ribosomal protein genes and throughout the rRNA gene locus in Saccharomyces cerevisiae. Mol Cell Biol 26: 3672-3679.

14. Lieb JD, Liu X, Botstein D, Brown PO (2001) Promoter-specific binding of Rap1 revealed by genome-wide maps of protein-DNA association. Nat Genet 28: 327-334.

15. Argimon S, Wishart JA, Leng R, Macaskill S, Mavor A, et al. (2007) Developmental regulation of an adhesin gene during cellular morphogenesis in the fungal pathogen Candida albicans. Eukaryot Cell 6: 682-692.

16. Rupp S, Summers E, Lo HJ, Madhani H, Fink G (1999) MAP kinase and cAMP filamentation signaling pathways converge on the unusually large promoter of the yeast FLO11 gene. Embo J 18: 1257-1269.

17. Hogues H, Lavoie H, Sellam A, Mangos M, Roemer T, et al. (2008) Transcription factor substitution during the evolution of fungal ribosome regulation. Mol Cell 29: 552-562.

18. Bastidas RJ, Heitman J, Cardenas ME (2009) The protein kinase Tor1 regulates adhesin gene expression in Candida albicans. PLoS Pathog 5: e1000294.

19. Harcus D, Nantel A, Marcil A, Rigby T, Whiteway M (2004) Transcription profiling of cyclic AMP signaling in Candida albicans. Mol Biol Cell 15: 4490-4499.

20. Hardwick JS, Kuruvilla FG, Tong JK, Shamji AF, Schreiber SL (1999) Rapamycin-modulated transcription defines the subset of nutrient-sensitive signaling pathways directly controlled by the Tor proteins. Proc Natl Acad Sci U S A 96: 14866-14870.

21. Jorgensen P, Rupes I, Sharom JR, Schneper L, Broach JR, et al. (2004) A dynamic transcriptional network communicates growth potential to ribosome synthesis and critical cell size. Genes Dev 18: 2491-2505.

22. Li H, Tsang CK, Watkins M, Bertram PG, Zheng XF (2006) Nutrient regulates Tor1 nuclear localization and association with rDNA promoter. Nature 442: 1058-1061.

23. Tsang CK, Bertram PG, Ai W, Drenan R, Zheng XF (2003) Chromatin-mediated regulation of nucleolar structure and RNA Pol I localization by TOR. Embo J 22: 6045-6056.
